# Characterization of a Novel Mouse Model of Alzheimer’s Disease—Amyloid Pathology and Unique β-Amyloid Oligomer Profile

**DOI:** 10.1371/journal.pone.0126317

**Published:** 2015-05-06

**Authors:** Peng Liu, Jennifer B. Paulson, Colleen L. Forster, Samantha L. Shapiro, Karen H. Ashe, Kathleen R. Zahs

**Affiliations:** 1 Department of Neurology, University of Minnesota, Minneapolis, Minnesota, United States of America; 2 University of Minnesota Academic Health Center Biological Materials Procurement Network (BioNet), University of Minnesota, Minneapolis, Minnesota, United States of America; 3 N. Bud Grossman Center for Memory Research and Care, University of Minnesota, Minneapolis, Minnesota, United States of America; 4 Department of Neuroscience, University of Minnesota, Minneapolis, Minnesota, United States of America; 5 Geriatric Research Education and Clinical Centers, Veterans Affairs Medical Center, Minneapolis, Minnesota, United States of America; Nathan Kline Institute and New York University Langone Medical Center, UNITED STATES

## Abstract

Amyloid plaques composed of β-amyloid (Aβ) protein are a pathological hallmark of Alzheimer’s disease. We here report the generation and characterization of a novel transgenic mouse model of Aβ toxicity. The rTg9191 mice harbor a transgene encoding the 695 amino-acid isoform of human amyloid precursor protein (APP) with the *Swedish* and *London* mutations (APP_NLI_) linked to familial Alzheimer’s disease, under the control of a tetracycline-response element, as well as a transgene encoding the tetracycline transactivator, under the control of the promoter for calcium-calmodulin kinase IIα. In these mice, APP_NLI_ is expressed at a level four-fold that of endogenous mouse APP and its expression is restricted to forebrain regions. Transgene expression was suppressed by 87% after two months of doxycycline administration. Histologically, we showed that (1) Aβ plaques emerged in cerebral cortex and hippocampus as early as 8 and 10.5-12.5 months of age, respectively; (2) plaque deposition progressed in an age-dependent manner, occupying up to 19% of cortex at ~25 months of age; and (3) neuropathology—such as abnormal neuronal architecture, tau hyperphosphorylation and misfolding, and neuroinflammation—was observed in the vicinity of neuritic plaques. Biochemically, we determined total Aβ production at varied ages of mice, and we showed that mice produced primarily fibrillar Aβ assemblies recognized by conformation-selective OC antibodies, but few non-fibrillar oligomers (e.g., Aβ*56) detectable by A11 antibodies. Finally, we showed that expression of the tetracycline transactivator resulted in reduced brain weight and smaller dentate-gyrus size. Collectively, these data indicate that rTg9191 mice may serve as a model for studying the neurological effects of the fibrillar Aβ assemblies *in situ*.

## Introduction

Beta-amyloid (Aβ) plaques are one of the pathological hallmarks of Alzheimer’s disease (AD). Aβ plaques can typically be classified as diffuse or dense-core types based on morphology and affinity for thioflavin S or Congo red [1]. It is well established that, in the brains of AD patients and amyloid precursor protein (APP) transgenic mice, dense-core—but not diffuse—plaques are associated with varied forms of neuropathology, such as neuroinflammation, abnormal neuronal architecture, and hyperphosphorylation and misfolding of tau [2–10] (for review see [1]). However, recent studies suggest that soluble oligomeric assemblies of Aβ are more likely responsible for AD pathogenesis (reviewed in [11, 12]). In AD patients or model organisms of the disease, Aβ assumes multiple oligomeric forms, including but not limited to: Aβ dimers, Aβ*56, amylospheroids, and annular protofibrils [13–17]. These brain-derived oligomers have been identified based on size and composition, and each has a unique spatial and temporal expression pattern [14–20]. In addition, Aβ assemblies have been characterized based on their immunoreactivity to conformation-selective antibodies—OC, which recognizes an epitope found in Aβ fibrils and some soluble oligomers (“fibrillar oligomers”), and A11 which recognizes a mutually exclusive epitope found on other soluble oligomers (“non-fibrillar oligomers”) [19–21].

Understanding the neurological effects of each type of oligomer has been difficult, because numerous Aβ assemblies often coexist within an individual brain. Here, we report the generation and characterization of a novel APP transgenic mouse model (rTg9191) uniquely suited to study the neurological effects of fibrillar Aβ aggregates *in situ*. These mice, which express a regulatable APP transgene, develop an amyloid plaque load similar to that of AD patients, and produce fibrillar Aβ assemblies but negligible amounts of non-fibrillar oligomers.

## Results

### Expression and suppression of APP transgene

**Fig 1:**
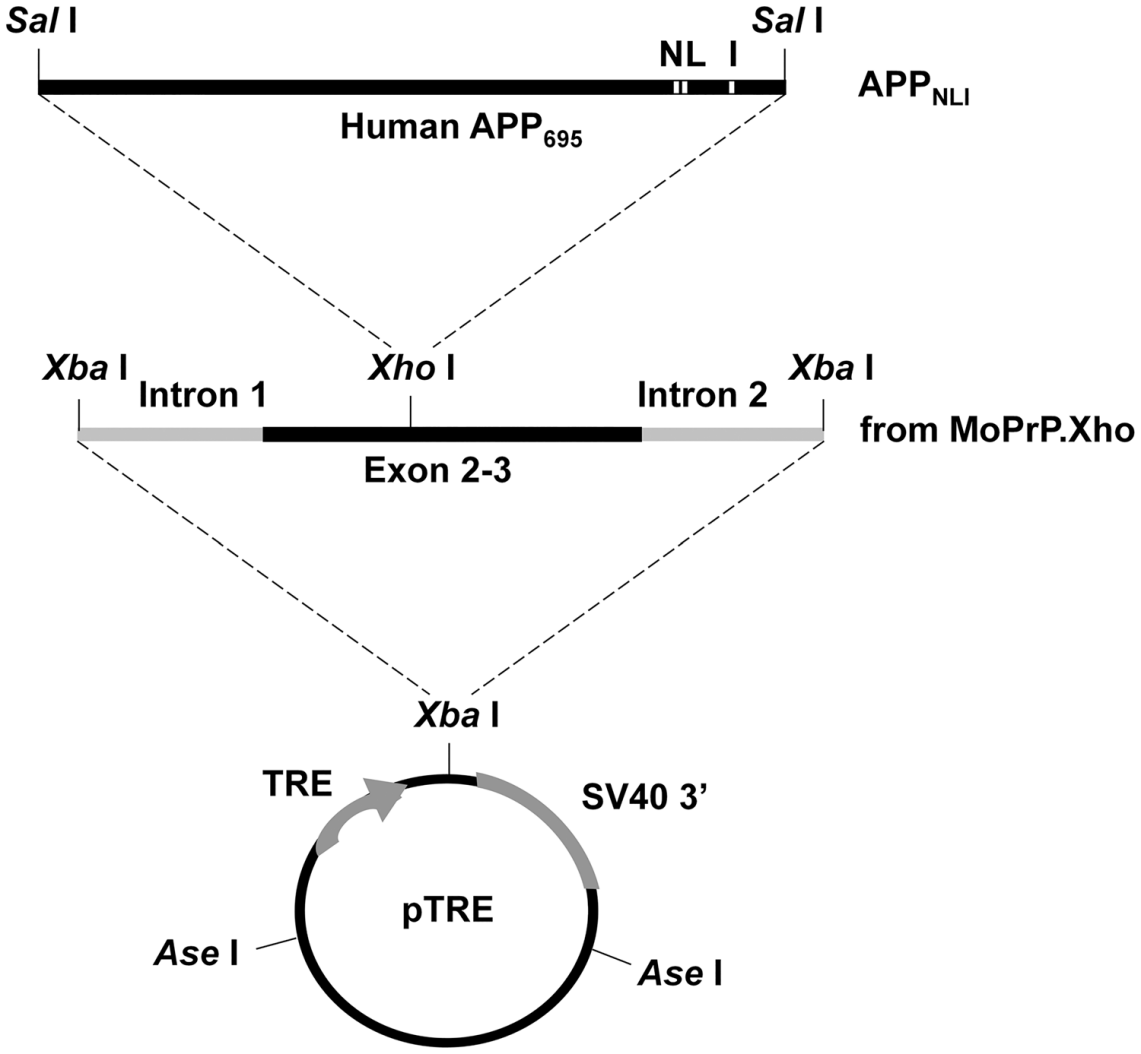
The APP_NLI_ responder transgene construct. The 695 amino acid-long amyloid precursor protein cDNA harboring the *Swedish* mutation (APP_NL_695) was inserted into the *Xho*I site of MoPrP.Xho fragment, which was further excised at two *Xba*I sites. The resulting fragment of prnp.APP_NL_ was cloned into the unique *Xba*I site in the inducible expression vector pTRE. The *London* mutation (V717I) was further introduced into the pTRE.prnp.APP_NL_ plasmid using site-directed mutagenesis.

**Fig 2:**
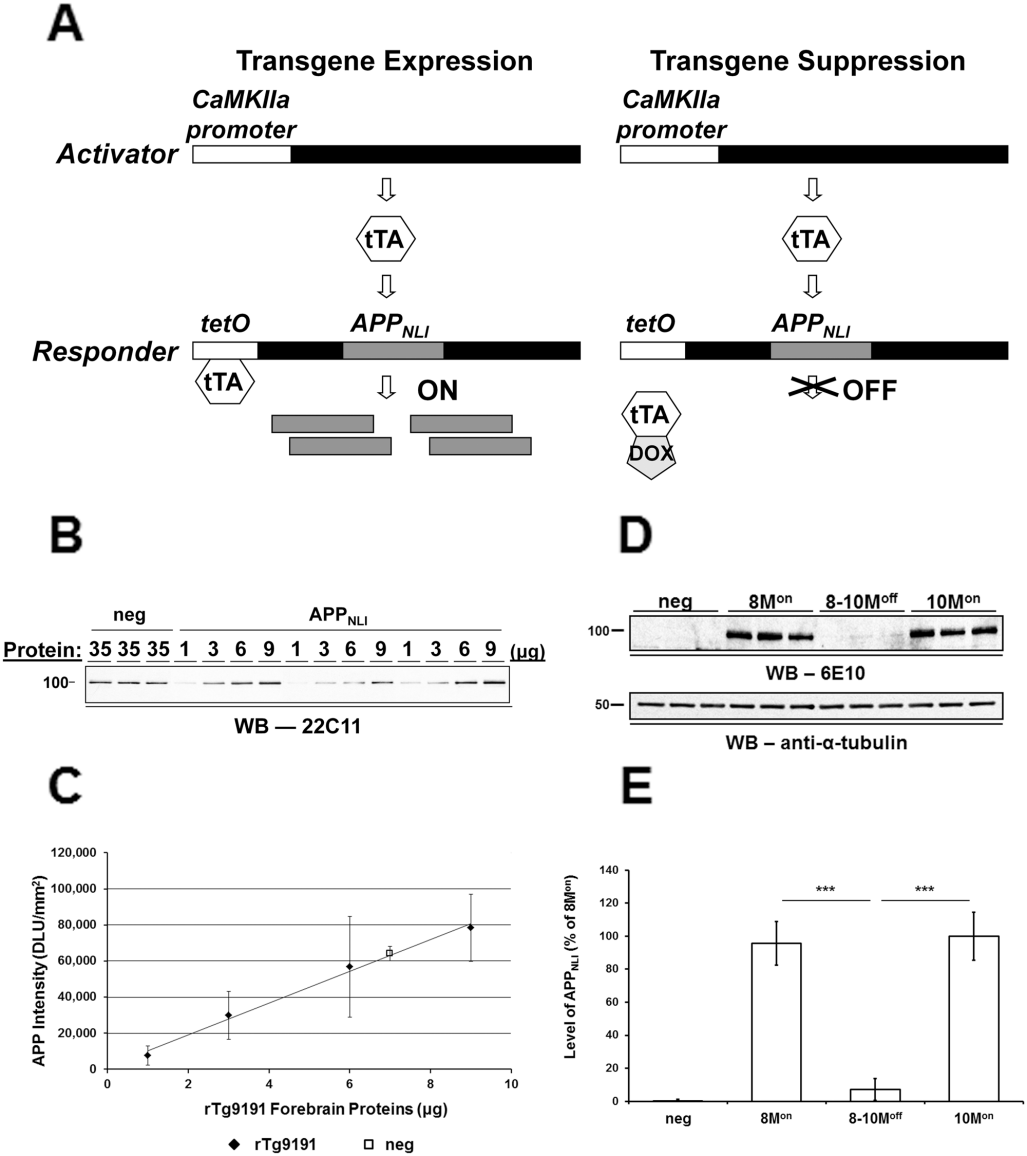
Expression and suppression of transgenic APP in rTg9191 mice. (A) Bigenic activator-repsonder system. rTg9191 mice employ a bigenic system in which a calcium-calmodulin kinase IIα (CaMKIIα) protomer drives constitutive expression of the tetracycline-controlled transactivator (*tTA*) gene, and a responder transgene for human APP_695_ containing the *Swedish* and *London* mutations (APP_NLI_) is under control of the tetracycline response element (*tet*O). Regulatable expression of the APP transgene in the rTg9191 line is under the control of doxycycline (DOX). In the absence of DOX, tTA binds the *tet*O promoter and APP_NLI_ is expressed; in the presence of DOX, the tTA-*tet*O interaction is blocked, and expression of APP_NLI_ is suppressed. (B-C) Expression of APP_NLI_. (B) Representative immunoblot probed with monoclonal antibody 22C11, which recognizes both mouse and human APP; numbers above the blot show amounts of protein loaded in each lane. (C) Quantification. Thirty-five μg of protein from brains of of 2-month-old non-transgenic (neg) mice is required to produce the same APP signal as 7 μg of protein from age-matched rTg9191 littermates, indicating that transgenic mice have 5 times more APP (mouse + human) than non-transgenic mice. Therefore, rTg9191 mice express 4 times more APP_NLI_ relative to mouse APP. DLU, densitometric light unit. (D-E) Suppression of APP_NLI_ expression. (D) Representative immunoblot using monoclonal antibody 6E10, which recognizes human Aβ1–16; 10 μg of protein was loaded in each lane. Alpha-tubulin served as the loading control. 8M^on^ and 10M^on^: 8- and 10-month-old rTg9191 mice without DOX treatment; 8-10M^off^: 10-month-old rTg9191 mice, treated with DOX from 8 to 10 months of age. (E) Quantification. Administration of DOX (200 ppm in chow) to rTg9191 mice decreased levels of APP_NLI_ by 87%. *** *p* < 0.0001, one-way ANOVA followed by Fisher’s *post hoc* analysis.

We created a regulatable transgenic mouse line, rTg9191, modeling Aβ pathology in AD. The rTg9191 mice harbor the 695 amino-acid isoform of human APP (APP_NLI_) with the *Swedish* (K670N and M671L, numbered according to the 770-amino acid isoform of APP) and *London* (V717I) mutations that are linked to familial AD (Fig 1). Expression of APP_NLI_ is driven by an interaction between the *tet*O promoter and the tetracycline-controlled transactivator (tTA), whose expression is driven by a calcium-calmodulin kinase IIα (CaMKIIα) protomer. Transgene expression can be suppressed by doxycycline (DOX)-mediated disruption of the tTA-*tetO* interaction (Fig 2A). Western blot analyses of the membrane-enriched fraction of brain protein extracts showed that the rTg9191 line expresses APP_NLI_ at a level four times that of endogenous mouse APP (Fig 2B and 2C). Additionally, we found that the level of APP_NLI_ remains constant with age and is two-thirds the transgenic APP level in Tg2576 mice (S1A and S1B Fig).

Administration of DOX to mice for 2 months resulted in an 87% reduction in the level of APP_NLI_ expression (Fig 2D and 2E).

### Regional expression pattern of APP transgene

**Fig 3:**
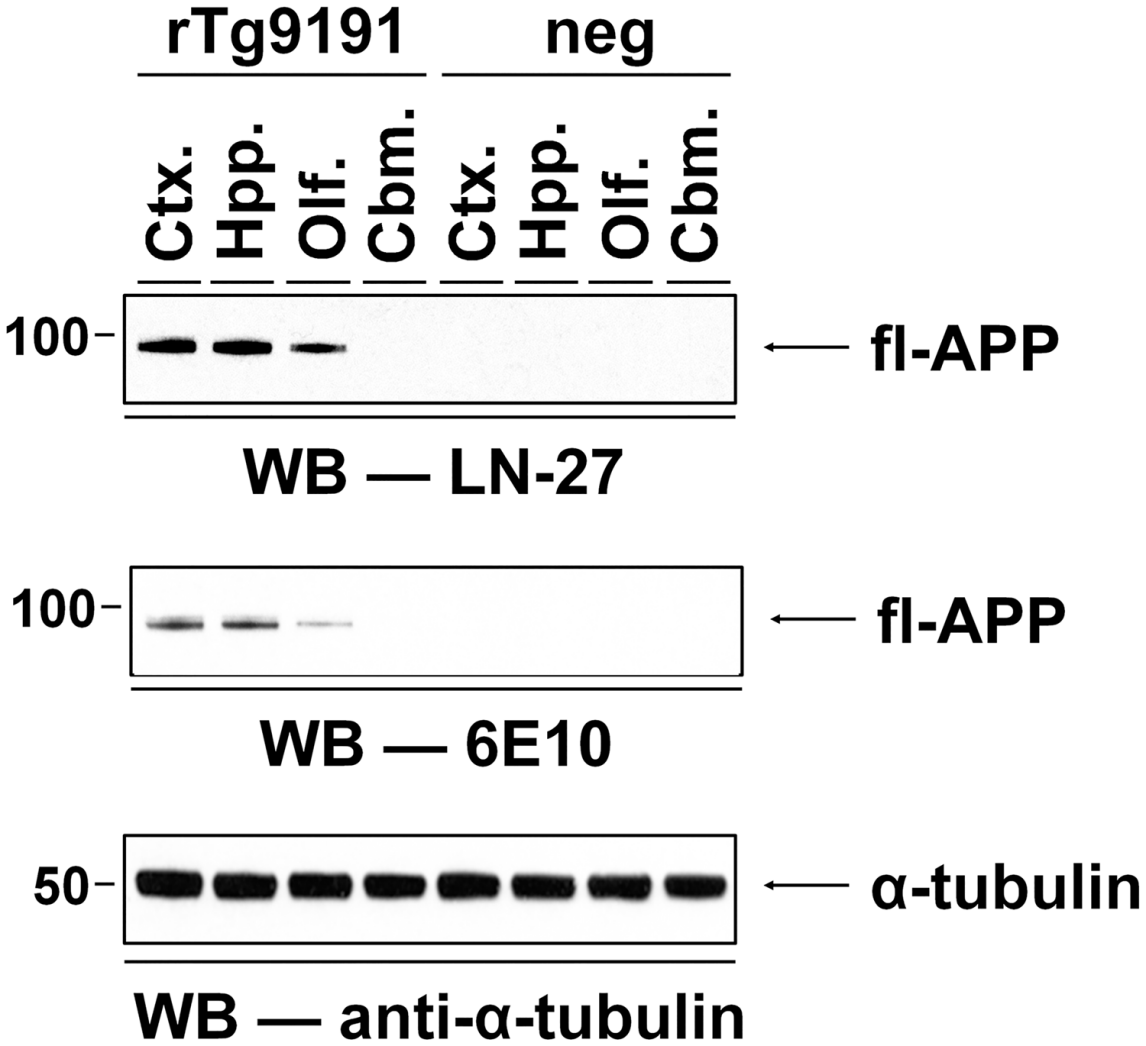
Regional expression pattern of the APP transgene in rTg9191 mice. The regional pattern of APP_NLI_ expression in four distinct anatomical structures (cerebral cortex (Ctx.), hippocampus (Hpp.), olfactory bulb (Olf.), and cerebellum (Cbm.)) of brain was analyzed using mouse monoclonal antibody LN27, which specifically recognizes human APP, and 6E10. The APP transgene was expressed in cerebral cortex and hippocampus with a minor portion in olfactory bulb; however, no expression was observed in cerebellum. No immunoreactivity using these human-specific antibodies was seen in non-transgenic littermates (neg). Alpha-tubulin served as the loading control. Representative blots show the APP_NLI_ expression pattern of female mice, and similar results were found in male mice.

The expression of APP_NLI_ in various brain regions was analyzed using LN27 and 6E10, two antibodies that recognize the N-terminus and Aβ region of human APP, respectively. For both antibodies, APP_NLI_ expression was observed in cerebral cortex, hippocampus, and olfactory bulb—but not in cerebellum; in addition, the level of expression was qualitatively similar in the cerebral cortex and hippocampus, but was lower in the olfactory bulb (Fig 3). This regional expression pattern of the transgene in rTg9191 mice is consistent with that seen in the rTg4510 and rTg3696AB lines, which share the same binary tet-off induction system as the rTg9191 line [22, 23].

### Beta-secretase-mediated APP processing

Beta-secretase-mediated digestion of APP to release C-terminal fragments (CTFβ) is the first step in amyloidogenic Aβ production. This 99 amino acid-long APP fragment is associated with multiple neurological ill-effects, including neuroinflammation and neurodegeneration, disruption of neuronal ionic homeostasis, and learning and memory impairments (reviewed in [24]). We measured the levels of CTFβ at different ages in rTg9191 mice and found an age-dependent increase in the level of CTFβ (S1A and S1C Fig), despite the fact that the level of APP_NLI_ remained constant with age. We also compared the levels of CTFβ in rTg9191 mice to the level found in Tg2576 mice. At 21 months of age, rTg9191 mice generate a level of CTFβ (relative to transgenic APP) equivalent to that of age-matched Tg2576 mice (S1A and S1C Fig), as might be expected, since both lines harbor the *Swedish* mutation.

### Age-dependent progression of Aβ plaques

**Fig 4:**
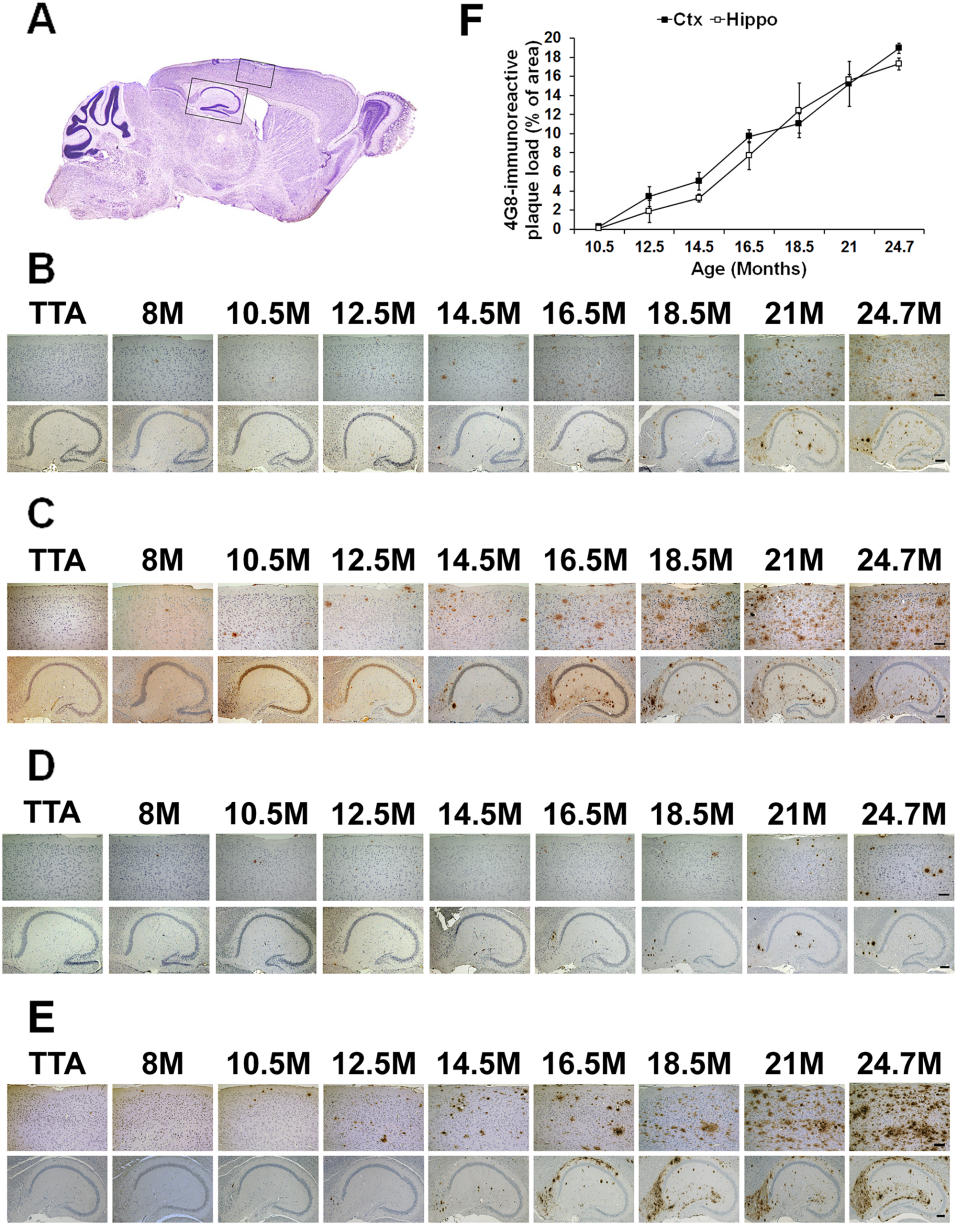
Age-related Aβ plaque progression in rTg9191 mice. (A) A representative sagittal section of brain. Black rectangles indicate the regions of cerebral cortex and hippocampal formation in which Aβ plaques are shown in (B-E). (B-E) Representative photomicrographs showing age-dependent progression of Aβ plaques in female mice, visualized using 6E10 (B); 4G8, directed against a mid-region of Aβ (C); 139–5, an Aβ_x-40_-specific antibody (D); and 1-11-3, an Aβ_x-42_-specific antibody (E). Upper panels, cerebral cortex; lower panels, hippocampus. Scale bars: 100 μm (upper panels), 200 μm (lower panels). TTA, mice expressing only the tetracycline transactivator. (F) Quantification of 4G8-immunoreactive Aβ plaque load at various ages.

We tracked the onset and accumulation of Aβ plaques in cerebral cortex and hippocampus of rTg9191 mice from 2 to 26 months of age. Plaques were visualized using four antibodies: 6E10 (recognizes an N-terminal region of Aβ), 4G8 (recognizes the mid- region of Aβ), 139–5 (Aβ40 end-specific antibody), and 1-11-3 (Aβ42 end-specific antibody). For all four antibodies, we found that plaques emerged first in the cerebral cortex, as early as 8 months of age, and then appeared in the hippocampus, between 10.5–12.5 months of age; plaque accumulation age-dependently progressed in both cortex and hippocampus (Fig 4A–4E). While 6E10, 4G8, and 1-11-3 detected both dense-core and diffuse plaque (Fig 4B, 4C and 4E; S2A, S2B, and S2D Fig), 139–5 seemed to recognize only dense-core plaques (Fig 4D; S2C Fig). We quantified burdens of 4G8-immunoreactive plaques in both cerebral cortex and hippocampus of mice between 10.5 and 24.7 months of age. These plaques included both the dense-core and diffuse types. Comparable plaque loads in cortex and hippocampus were found at each of the ages examined. At 24.7 months of age, plaques loads reached 19% and 17%, respectively, for cortex and hippocampus (Fig 4F). We also revealed dense-core plaques of aged rTg9191 mice using thioflavin S. At 24.7 months of age, plaque loads were 0.41% and 0.37% for cortex and hippocampus, respectively (S2E and S2F Fig).

### Age-related Aβ production

**Fig 5:**
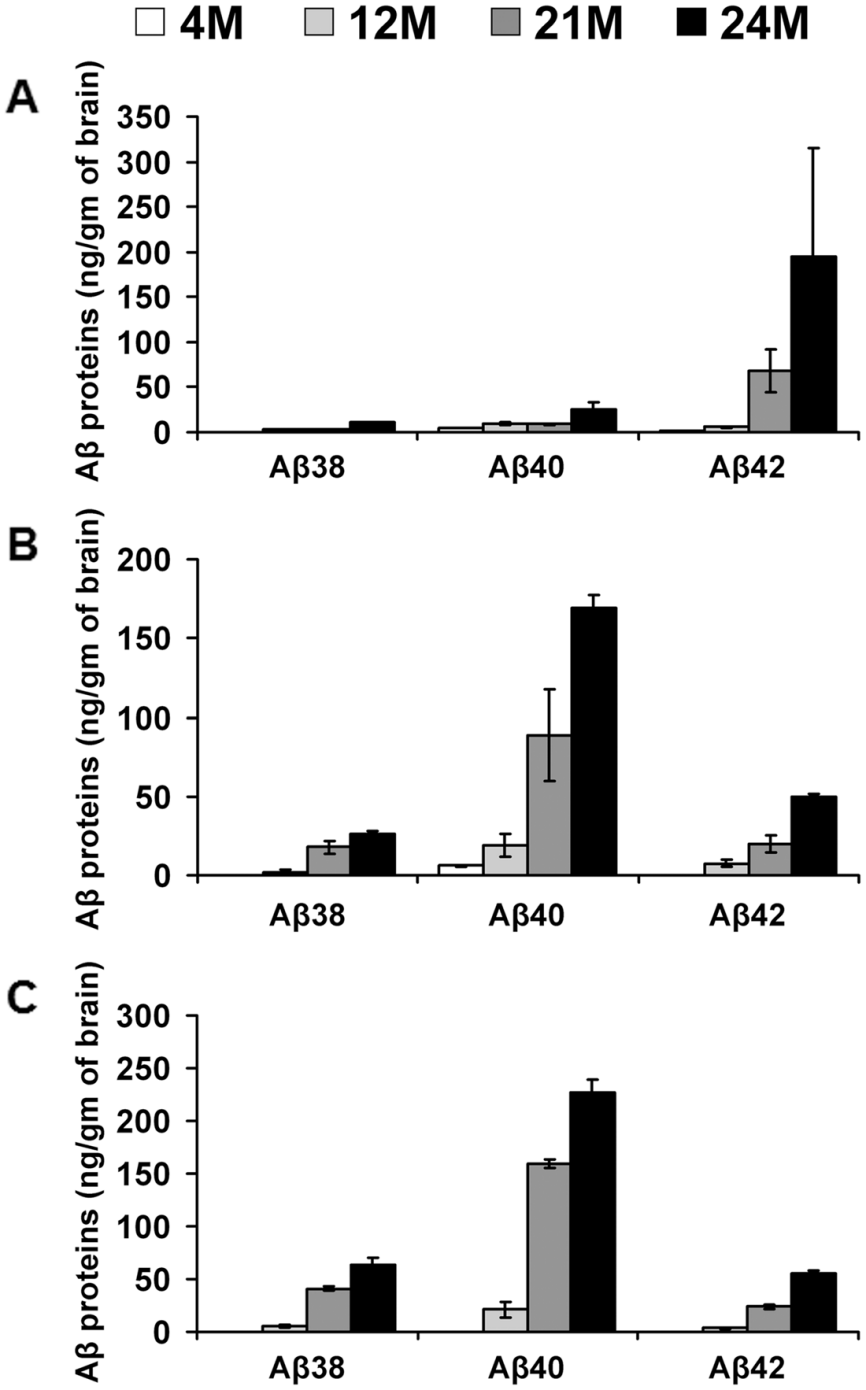
Age-related production of Aβ proteins in water-soluble, detergent-soluble, and detergent-insoluble fractions of brain extracts from rTg9191 mice. The levels of total Aβ38, Aβ40, and Aβ42 proteins in water-soluble (A), detergent-soluble (B) and detergent-insoluble (C) fractions of brain extracts of rTg9191 mice at 4, 12, 21, and 24 months of age were measured using ELISA.

We determined the levels of Aβ38, Aβ40, and Aβ42 proteins in brain parenchyma from rTg9191 mice at young (4 months), middle (12 months), and old (21 and 24 months) ages using an enzyme-linked immunosorbent assay (ELISA). The levels of Aβ proteins were separately measured in the water-soluble (Fig 5A), detergent-soluble (Fig 5B), and detergent-insoluble (Fig 5C) fractions. Overall, we observed an age-dependent increase in the production of Aβ38, Aβ40, and Aβ42 in all three fractions—the one exception being that the level of Aβ40 in the water-soluble fraction of 21-month-old mice was slightly lower than that of 12-month-old mice. This relative reduction in Aβ40 might be caused by the coincidental formation of Aβ40-comprising dense-core plaques (Fig 4D). Of particular interest is that in the water-soluble fraction, levels of Aβ40 were higher than Aβ42 prior to plaque formation (i.e., at 4 and 12 months of age), but, in aged mice (i.e., 21 and 24 months of age), this relationship between Aβ40 and Aβ42 was reversed (Fig 5A); in addition, in 21- and 24-month-old mice with high plaque loads, levels of Aβ42 decreased from water-soluble to detergent-soluble and insoluble fractions, whereas levels of Aβ40 increased (Fig 5A–5C). These findings, together with observations concerning age-related plaque progression in mice, indicate that a majority (> 90%) of Aβ40 builds the dense cores of plaques, and that Aβ42 accounts for the main Aβ components of diffuse plaques and loosely core-associated oligomers, which remain water-soluble following protein extraction.

### Age-related production of soluble Aβ oligomeric assemblies

**Fig 6:**
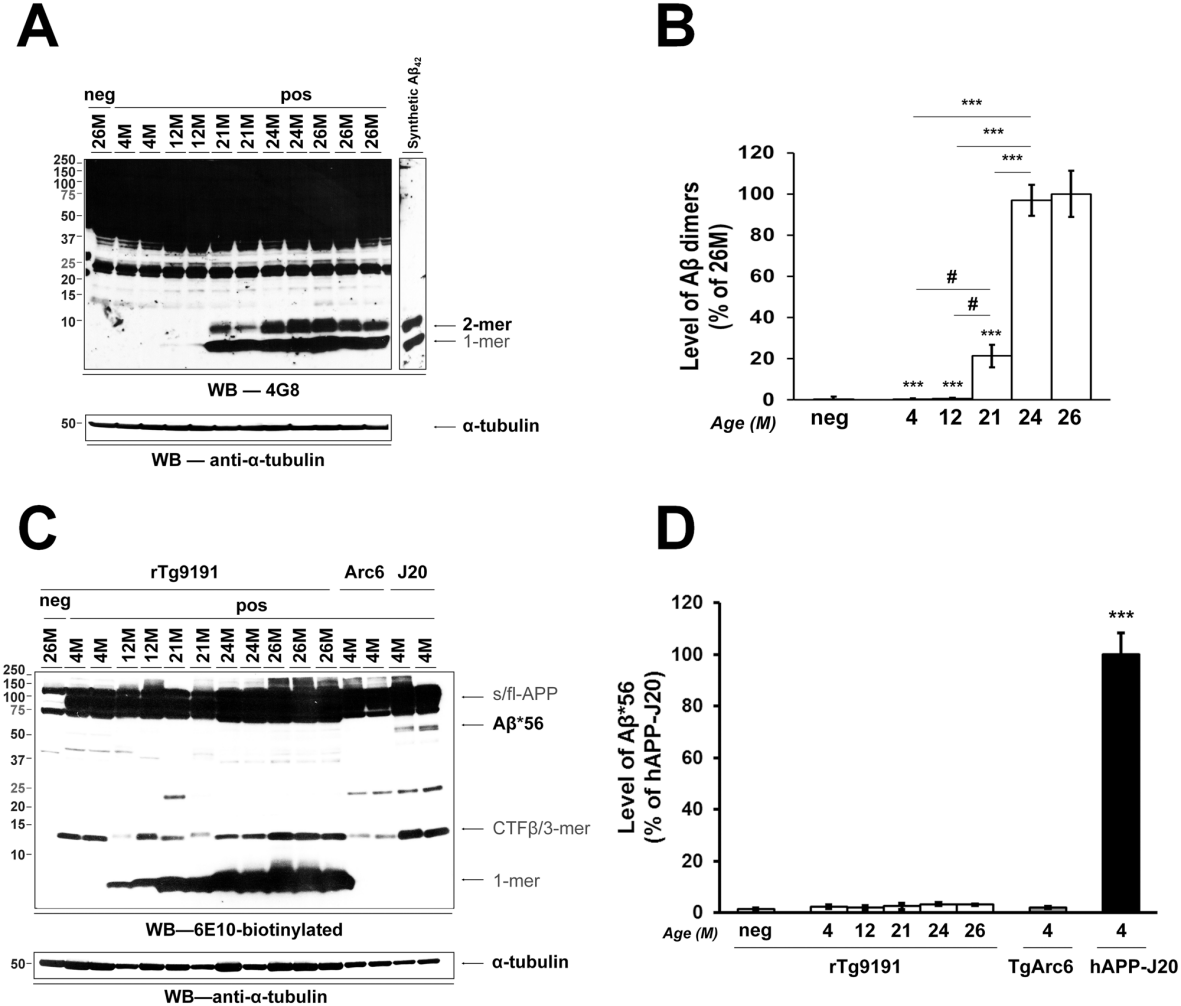
rTg9191 mice produce Aβ dimers in an age-dependent manner, but lack Aβ*56. (A-B) Production of Aβ dimers. (A) A representative immunoblot (upper panel) shows levels of Aβ dimers in young, mid-age, and old mice; α-tubulin served as the loading control (lower panel). (B) Quantification. rTg9191 mice exhibit an age-dependent progression in levels of Aβ dimers. # p < 0.05, *** p < 0.0001 (compared to 26M), one-way ANOVA, followed by Fisher’s *post hoc* analysis. (C-D) rTg9191 mice lack Aβ*56. (C) Representative immunoblot shows levels of Aβ*56 in transgenic mouse lines rTg9191, TgArc6, and hAPP-J20. Although a faint band at ~56kDa was seen occasionally in extracts from rTg9191 mice, the intensity of this band was comparable to that seen in some samples from non-transgenic mice. As monoclonal antibody 6E10 recognizes human, but not mouse Aβ, this faint band represents non-specific background noise, and not a true Aβ signal. (D) Quantification. The intensity of the ~56 kDa band in rTg9191 mice (4, 12, 21, 24 and 26M of age) is comparable to that of non-transgenic littermates (neg, 26M) and TgArc6 (4M), but is significantly lower than that of hAPP-J20 (4M). *** p < 0.0001, each group compared to hAPP-J20, one-way ANOVA, followed by Fisher’s *post hoc* analysis. Neg, non-transgenic littermates of rTg9191 mice; pos, mice that express APP transgenes. Samples from both genders were loaded onto the representative blots, aligning in the order of left to right for (A): F, M, F, F, M, M, F, M, F, M, F, F (M, male; F, female), and for (C): F, F, M, M, F, M, F, M, F, M, F, F, M, F, M, F.

We next investigated age-dependent production of soluble Aβ oligomeric assemblies in brains of rTg9191 mice. We asked whether rTg9191 mice produce Aβ dimers and Aβ*56, the two brain-derived oligomeric assemblies that have been linked to memory deficits and memory-related electrophysiological dysfunction [14, 15]. Under denaturing experimental conditions, we analyzed these oligomers from brain extracts of young (4 months of age), mid-aged (12 months of age), and old (21, 24, and 26 months of age) mice. Aβ dimers were not detected at 12 months of age; however from 21–24 months of age, dimer levels increased steeply and then plateaued (Fig 6A and 6B). In contrast, Aβ*56 was not detectable at any age in rTg9191 mice. Aβ*56 similarly was absent from memory-intact TgArc6 mice, but was prominent in memory-impaired 4-month-old hAPP-J20 mice [25] included for comparison (Fig 6C and 6D). Therefore, rTg9191 mice produce Aβ dimers in an age-dependent manner, but do not generate detectable levels of Aβ*56.

**Fig 7:**
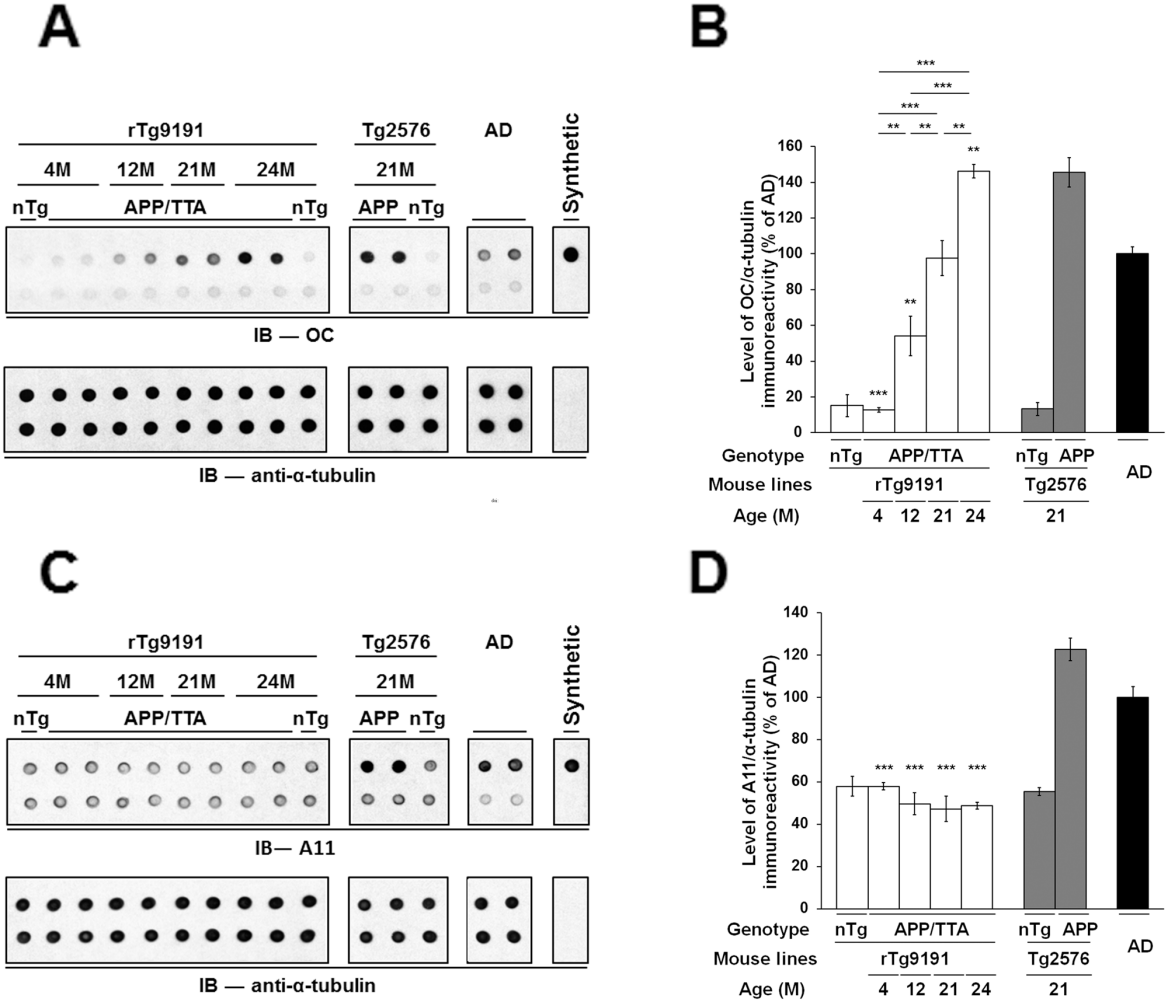
rTg9191 mice age-dependently produce soluble Aβ oligomers immunoreactive to OC but not to A11 antibodies. (A-B) Soluble OC-immunoreactive Aβ oligomers accumulate in brains of rTg9191 mice in an age-dependent manner. (A) Upper panels, representative dot blots showing levels of protein oligomers detected by polyclonal OC antibodies in water-soluble brain extracts from rTg9191 and Tg2576 mice and AD patients. Upper lane, brain extracts and synthetic aggregates; lower lane, brain extracts and synthetic Aβ aggregates immunodepleted of Aβ, using an array of antibodies (6E10, 4G8, 139–5, and 1-11-3). OC immunoreactivity disappears when samples are immunodepleted of Aβ, suggesting that the OC signals in the brain extracts arise from Aβ assemblies. Each dot contains either 0.5 μg of protein extracts or 2 ng of synthetic aggregates. Synthetic soluble Aβ aggregates with in-register parallel β-sheets were used as a positive control. Alpha-tubulin served as the loading control (lower panel) for both the untreated (upper lane) and Aβ-immunodepleted materials (lower lane). (B) Quantification. rTg9191 mice show an age-dependent increase in levels of OC immunoreactivity. At 21 months of age, rTg9191 mice have a comparable level of OC-immunoreactive signals to AD patients. ** p < 0.001, *** p < 0.0001, each group compared to AD, one-way ANOVA, followed by Fisher’s *post hoc* analysis. (C-D) rTg9191 mice lack A11-immunoreactive Aβ oligomers in brain. (C) Upper panels, representative dot blots showing levels of protein oligomers detected by polyclonal A11 antibodies in water-soluble brain extracts from rTg9191 and Tg2576 mice and AD patients. Upper lane, brain extracts and synthetic oligomers; lower lane, brain extracts and synthetic Aβ oligomers immunodepleted of Aβ, using an array of antibodies (6E10, 4G8, 139–5, and 1-11-3). Each dot contains either 1 μg of protein extracts or 1 μg of synthetic oligomers. Alpha-tubulin served as the loading control (lower panel) for both the untreated (upper lane) and Aβ-immunodepleted materials (lower lane). (D) Quantification. A11 immunoreactivities in brains of rTg9191 mice show no age-dependent change and are comparable to those of young and old non-transgenic littermates. Aged Tg2576 mice and AD patients, however, show significantly higher A11 immunoreactivity. *** p < 0.0001, each group compared to AD, one-way ANOVA, followed by Fisher’s *post hoc* analysis. nTg, non-transgenic littermates of rTg9191 or Tg2576 mice; APP/TTA, rTg9191 mice that harbor both the TTA activator and APP responder transgenes and therefore express APP_NLI;_ APP, Tg2576 mice that express the APP transgene. For each detection antibody, all panels in an image came from a single representative blot; blots are shown segmented for clarity. Samples from both genders were loaded onto the representative blots, aligning in the order of left to right for both (A) and (C): F, M, F, M, F, M, F, M, F, F, M, F, F, M, F (M, male; F, female).

We also employed dot blotting to characterize Aβ oligomers in the water-soluble fraction of brain extracts, under non-denaturing conditions. We used OC antibodies to examine and compare levels of soluble fibrillar oligomers in rTg9191 mice at various ages, aged Tg2576 mice, and AD patients (Fig 7A and 7B). We found that rTg9191 mice exhibited an age-dependent increase in OC immunoreactivity. At 4 months of age, OC immunoreactivity of rTg9191 mice was no more than that of non-transgenic controls. When rTg9191 mice reached 21 months of age, the level of OC immunoreactivity matched that of AD patients; at 24 months of age, OC immunoreactivity further increased and was comparable to that of 21-month-old Tg2576 mice. To confirm that the OC immunoreactivity arose from Aβ oligomers, we performed immunodepletion using an array of antibodies directed against various epitopes on Aβ. Immunodepleting Aβ from brains of rTg9191, Tg2576 mice, and AD patients decreased OC immunoreactivity to a level comparable to that of the non-transgenic brains, indicating that OC-immunoreactive signals come from soluble Aβ oligomers.

In parallel, we used A11 antibodies to detect soluble non-fibrillar oligomers in rTg9191 and Tg2576 mice, as well as in AD patients (Fig 7C and 7D). We found that levels of A11 immunoreactivity in rTg9191 mice at all ages were comparable to levels in non-transgenic littermates. In contrast, aged Tg2576 mice and AD patients exhibited higher A11 immunoreactivities. Immunodepletion of Aβ decreased A11 immunoreactivity in brains of both Tg2576 mice and AD patients, which have previously been shown to contain the A11-positive oligomer Aβ*56 [15, 18].

In summary, our results show that rTg9191 mice produce OC-immunoreactive fibrillar Aβ oligomers in an age-dependent manner, but have few, if any, non-fibrillar Aβ oligomers as recognized by A11 antibodies.

### TTA expression is associated with low brain weights and small dentate gyri

**Fig 8:**
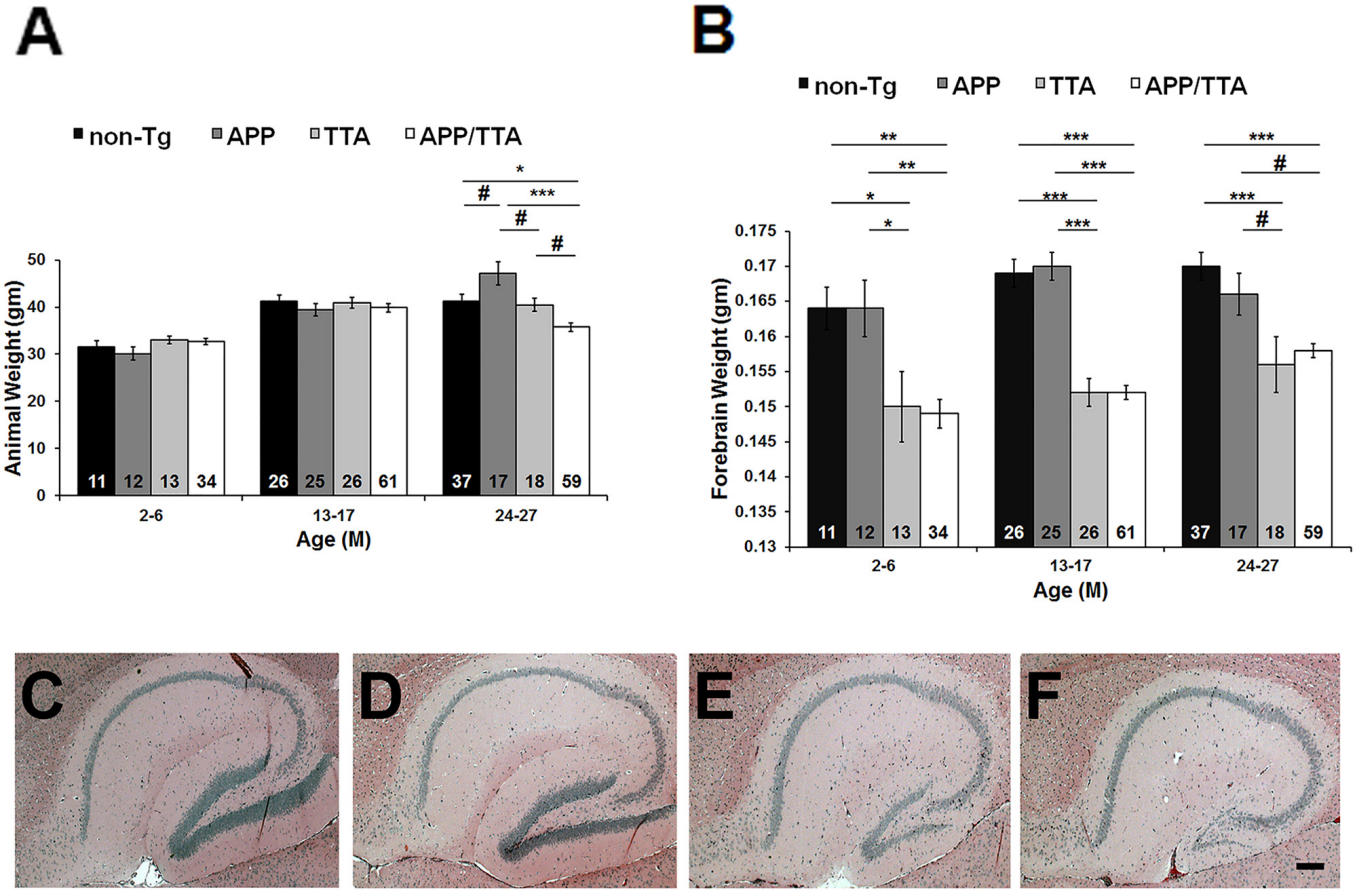
TTA expression results in reduced forebrain weight and dentate-gyrus size. (A) Weights of rTg9191 mice (APP/TTA), littermates harboring only the activator gene (TTA), only the responder gene (APP), and neither gene (non-Tg). There were no genotype-related differences in body weight in young and mid-aged mice. Aged rTg9191 mice, however, had lower body weights compared to their littermates. (B) rTg9191 mice and TTA littermates have lower forebrain weights than their APP and non-Tg littermates at all ages studied. The numbers of mice examined are shown for each genotype. # p < 0.05, * p < 0.01, ** p < 0.001, *** p < 0.0001, two-way ANOVA followed by Fisher’s *post hoc* analysis. The percentage of female mice in the genotype of APP/TTA, TTA, APP, and non-Tg is 50%, 38%, 67%, and 55%, respectively for the 2–6 month-old; 51%, 35%, 48%, and 50%, respectively for the 13–17 month-old; 46%, 61%, 47%, and 51%, respectively for the 24–27 month-old. Chi square/Fisher exact tests showed no significant difference in gender distribution between genotypes. (C-F) Representative photomicrographs showing hematoxylin and eosin staining of the hippocampal regions of 16.5-month-old rTg9191 mice and their age-matched littermates. Sections at ~1.20 mm lateral from the midline were used. The sizes of dentate gyri of rTg9191 (F) and TTA (E) mice are decreased compared to those of APP (D) and non-Tg (C) littermates. Scale bar: 200 μm, applies to C-F. Representative photomicrographs show hippocampus hematoxylin and eosin staining of female mice, and similar results were observed in male mice.

A previous study showed that expression of the tetracycline transactivator (TTA) during development resulted in neuron loss in the hippocampus of transgenic mice [26]. We investigated the effects of TTA expression on hippocampal size and brain weight. For this, we first compared body and brain weights of rTg9191 mice with their littermates carrying only the TTA transgene, only the APP_NLI_ transgene, or no transgenes. We grouped mice into three categories: young (2–6 months of age), middle-aged (13–17 months of age), and old (24–27 months of age) to increase the size of each group and enhance the power of statistical analysis. No genotype-related alteration in body weight was found in young and middle-aged mice, while old rTg9191 had lower body weights than the other three genotypes (Fig 8A). Forebrain weights of the TTA-expressing mice were significantly lower than mice expressing no TTA (Fig 8B).

To understand the structural basis of the reduction in brain weight induced by TTA expression, we examined the size of the hippocampus. Consistent with the previous report [26], we found that at middle age (~17 months of age), TTA-expressing mice (rTg9191 and TTA) had smaller dentate gyri and thinner granule cell layers, compared to non-transgenic littermates and mice harboring only the APP_NLI_ transgene (Fig 8C–8F). No genotype-related differences were noticeable in the CA1 and CA3 areas.

### Plaque-associated neuropathology

**Fig 9:**
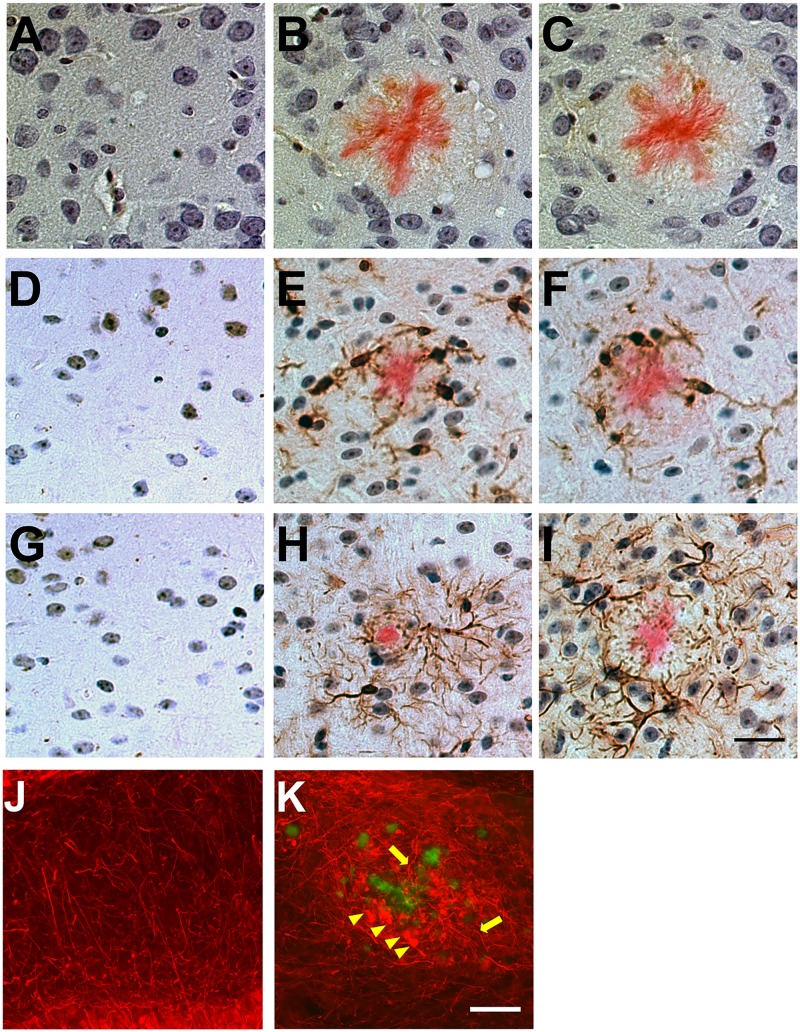
Plaque-associated neuroinflammation and abnormal neuronal architecture in rTg9191 mice. (A-I) rTg9191 mice show reactive gliosis in the vicinity of dense-core plaques. Brain sections from rTg9191 mice at 24 months of age (B, E, H), their age-matched non-transgenic littermates (A, D, G), and age-matched Tg2576 mice (C, F, I) were stained with antibodies directed against the astroglial marker S100β (A-C), a monoclonal antibody directed against the microglial marker ionized calcium-binding adaptor molecule 1 (Iba1) (D-F), and an antibody directed against the astrocytic marker glial fibrillary acidic protein (GFAP) (G-I). Astrocytes and activated microglial cells and reside near dense-core plaques visualized using Congo red (pink). Scale bar in I, 25 μm, applies to A-I. (J-K) rTg9191 mice exhibited abnormal neuronal architecture around plaques. Thioflavin S (green) was used to visualize plaques and monoclonal antibody SMI-312 was used to visualize axons (red). (J) No plaques were detected in age-matched non-transgenic littermates of rTg9191 mice, and neuronal morphologies were normal. (K) Plaques are surrounded by swollen, dystrophic axons (arrowheads) and curvy, distorted axonal processes (arrows) in brains of rTg9191 mice. Scale bar in K, 50 μm, applies to J and K. Representative photomicrographs show neuroinflammation and neuronal architecture of female mice, and similar results were found in male mice.

**Fig 10:**
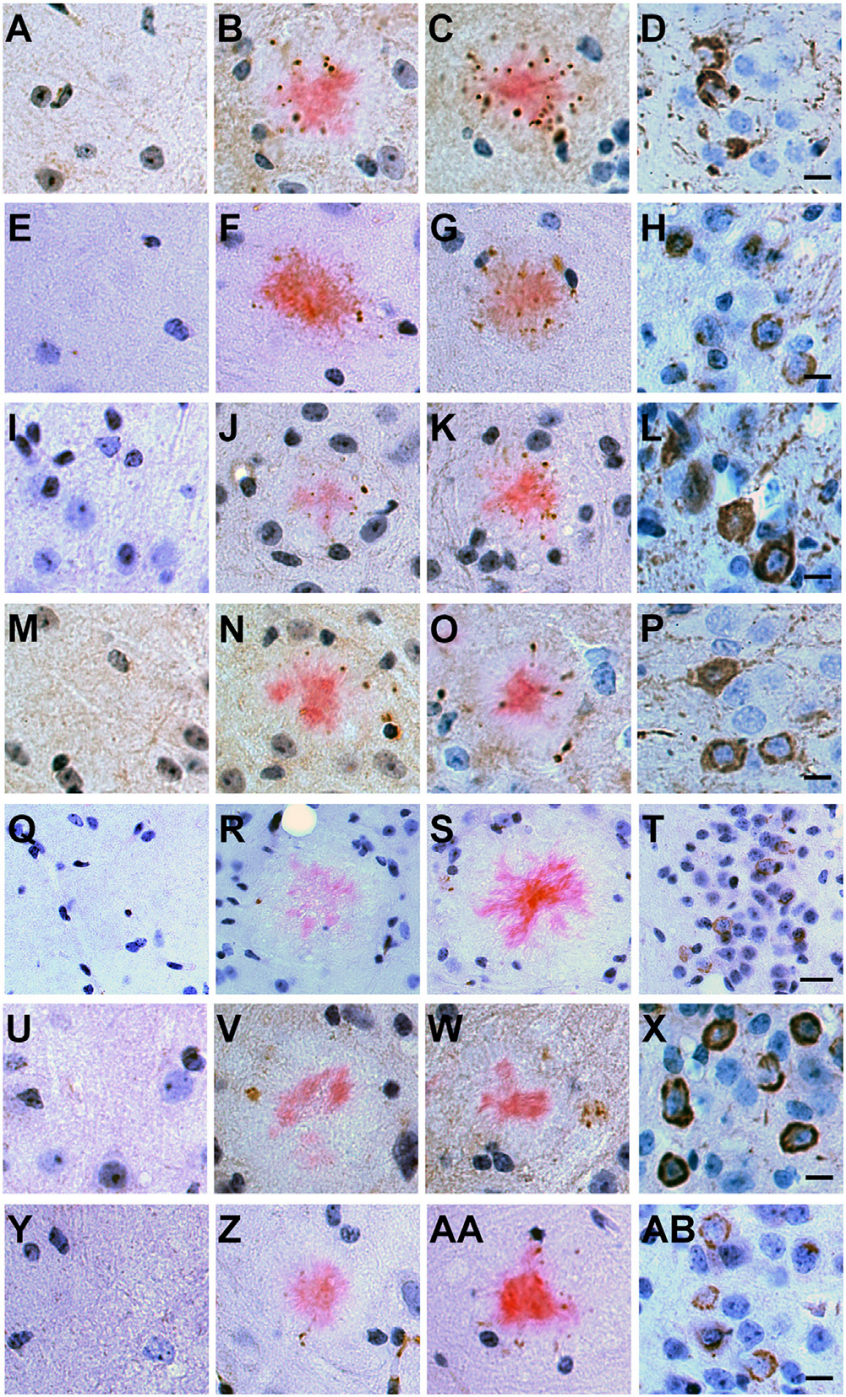
Plaque-associated tau pathology in rTg9191 mice. Brain sections from rTg9191 mice of 24 months of age (B, F, J, N, R, V, Z), their age-matched non-transgenic littermates (A, E, I, M, Q, U, Y), 23-month-old Tg2576 mice (C, G, K, O, S, W, AA) and 15-month-old rTg4510 mice (D, H, L, P, T, X, AB) were stained with a variety of antibodies directed against pathological conformation- and phosphorylation-dependent epitopes of tau: AT8 (A-D), CP13 (E-H), PG5 (I-L), PHF-1 (M-P), Alz50 (Q-T), MC1 (U-X) and TG-3 (Y-AB). Representative photomicrographs showed that hyperphosphorylated and/or misfolded tau proteins accumulated (brown puncta) around dense-core plaques visualized using Congo red (pink). Neuronal staining (brown) in rTg4510 mice served as positive control. Scale bars: 20 μm. Images in the same row have the same magnification. Representative photomicrographs show tau pathology of female mice, and similar results were found in male mice.

We asked whether rTg9191 mice exhibit neuropathology in the vicinity of plaques, as has been described in the brains of other APP transgenic mice and in AD patients [5, 7, 10, 27]. We found that, similar to Tg2576 mice, gliosis in rTg9191 was associated with Congo red-positive, dense-core plaques (Fig 9A–9I). In addition, we found aggravated axonal curvature and swollen, dystrophic neurites surrounding thioflavin S-positive plaques in rTg9191 mice (Fig 9J and 9K), resembling findings in AD and transgenic mouse brains [5, 10]. We next examined plaque-associated tau pathology using an array of well-characterized antibodies directed against hyperphosphorylated and conformationally altered tau forms (Fig 10). Immunoreactive profiles surrounded dense-core plaques in rTg9191 mice, as also shown in Tg2576 mice (this study, [28]).

To rule out the possibility that plaque-associated neuropathology was induced by the expression of tetracycline transactivator (TTA), we examined these pathological features in mice expressing only TTA. We showed that similar to the non-transgenic, no gliosis, neuronal dystrophy or tau hyper-phosphorylation was observed in brains of rTg9191 littermates expressing only TTA (S3 and S4 Figs).

## Discussion

In this report, we describe the generation and the biochemical and immunohistochemical characteristics of a regulatable APP transgenic mouse model—rTg9191.

We used sequence-specific antibodies to determine the temporal profiles of individual Aβ oligomers detected under denaturing conditions, and found that levels of Aβ dimers increased in an age-dependent manner, but that Aβ*56 was absent from rTg9191 mice. In addition, we used conformation-selective antibodies to detect Aβ assemblies under non-denaturing conditions. Studies using conformation-selective antibodies indicate that there are at least two structurally distinct classes of amyloid oligomers: fibrillar assemblies and non-fibrillar assemblies, recognized by the polyclonal antibodies OC and A11, respectively [29]. The OC antibodies preferentially recognize the in-register parallel β-sheet structure (Liu et al. manuscript submitted) that full-length Aβ adopts in neuritic plaques of AD brain [30]. The exact conformation that is detected by A11 antibodies is currently unresolved, but there is evidence that A11 recognizes an out-of-register, anti-parallel β-sheet structure [31, 32]. A unique feature of rTg9191 mice is that they produce primarily fibrillar Aβ assemblies, and have few, if any, non-fibrillar oligomers that are immunoreactive to A11.

We showed here that an age-dependent increase in OC-immunoreactive Aβ assemblies paralleled the age-dependent increase in dimer levels. This observation begs the question of whether Aβ dimers, detected under denaturing conditions on Western blots, are related to OC-immunoreactive fibrillar oligomers, detected under native conditions using dot blots. Additional biochemical experiments suggest that Aβ dimers might represent breakdown products of larger OC-positive oligomers. When water-soluble brain extracts of aged rTg9191 mice were fractionated using size exclusion chromatography (SEC), a majority of Aβ proteins were eluted in two peaks with apparent molecular weights of 670–2,000 kDa and ~150 kDa. Upon Western blot analysis, both SEC peaks were shown to contain Aβ dimers, indicating that either (a) these dimers may arise from disassembled high-molecular-weight OC-immunoreactive assemblies, or (b) that free dimers do exist in the brain, but are eluted anomalously because of their possible non-globular structure (Liu et al. manuscript submitted).

The generation of a transgenic mouse line that specifically generates fibrillar Aβ aggregates was fortuitous. The particular Aβ oligomer profile generated in each line of APP transgenic mouse likely results from multiple factors, including genetic background, level of transgene expression, and Alzheimer’s disease-linked mutation(s) in the transgene that influence the Aβ42/Aβ40 ratio. APP processing, Aβ metabolism and amyloid deposition have been shown to vary with genetic background, even when levels of APP expression remain constant [33]. Studies comparing hAPP-J20, TgArc6, and TgArc48 mice suggest that both the levels of transgene expression and the propensity for aggregation of the resulting A peptides are also critical factors governing the levels of globular oligomers, such as Aβ*56 [25]. hAPP-J20 mice carry an APP mini-gene with *Swedish* ((K_670_M_671_ → N_670_L_671_) and *Indiana* (V_717_ → F_717_) mutations, while TgArc6 and TgArc48 mice carry this same transgene with the addition of the *Arctic* (E_693_ → G_693_) mutation; all three lines are in the same C57Bl/6 genetic background. A comparison of TgArc48 and TgArc6 mice suggests that APP levels influence Aβ oligomer formation: although these two lines carry the same APP transgene in the same genetic background, TgArc48 mice express six times more APP and three times more Aβ*56 than do TgArc6 mice. Further, comparing TgArc mice to hAPP-J20 mice suggests that the fibrillogenicity of the Aβ peptides also influences the oligomer profile: the lines bearing the *Arctic* mutation generate approximately half the amount of Aβ*56, normalized to APP levels, as does the hAPP-J20 line. Cheng and colleagues hypothesized that accelerating the formation of fibrils, by inclusion of the *Arctic* mutation, may lower the globular Aβ species either by diverting monomers in a limiting pool away from the formation of globular assemblies, or by sequestering the globular assemblies directly into fibrils [25].

The APP transgene in rTg9191 mice is similar to that in hAPP-J20 mice in that it contains the *Swedish* mutation that increases production of all forms of A [34, 35], as well as an additional mutation (the *Indiana* mutation in hAPP-J20 and the *London* mutation (V_717_ → I_717_) in rTg9191) that shifts APP processing towards Aβ42 [36] (reviewed in [37]). However, hAPP-J20 mice generate Aβ*56, while rTg9191 mice do not. At this time, we can only speculate about which factors account for the different oligomer profiles observed in these two lines. Not only do rTg9191 and hAPP-J20 mice differ in genetic background, but also in the construction of the APP transgene. As described here, rTg9191 mice are bi-genic, with the CaMKIIα promoter driving expression of the tetracycline transactivator in excitatory neurons of the forebrain [38], while the responder consists of cDNA encoding the 695-amino acid isoform of APP. By contrast, hAPP-J20 mice carry an APP mini-gene driven by the platelet-derived growth factor promoter [39]. Notably, the two lines differ in the amount of Aβ produced, in an age-dependent manner. At younger ages, steady-state levels of soluble Aβ40 and Aβ42 are comparable in hAPP-J20 and rTg9191 mice, but in mice greater than a year of age, levels of Aβ peptides in rTg9191 mice greatly exceed those measured in hAPP-J20 mice (compare values shown in Fig 5 of this report to Fig 2 of [40]).

In addition to examining plaque-associated neuropathology, we also evaluated the relevance of our novel model to AD and several other mouse models of AD by quantifying and comparing dense-core plaque load in brain parenchyma. We observed that the density of neuritic plaques in rTg9191 mice was comparable to that in AD brains, whereas in some other mouse model systems, such as *Tg(APPswe*,*PSEN1dE9)85* [25] and *Tg(5xFAD)6799* [41], plaque loads were substantially greater than in AD (Liu et al. manuscript submitted).

In summary, we have developed rTg9191 mice, a novel regulatable APP transgenic model that specifically produces fibrillar Aβ assemblies. This unique feature allows studying the neurological effects of fibrillar Aβ assemblies *in situ*, including the effects of such assemblies on cognition and plaque-associated neuropathology. Further, the regulatable property of the model allows temporal modulation of APP expression and Aβ production. This feature enables studies of the interactions of Aβ with age-related factors and, of particular clinical relevance, studies of the persistence of Aβ-triggered pathology following reductions in Aβ production.

## Materials and Methods

### Generation of rTg9191 mice

Our methods for generating rTg9191 mice utilized a binary system of responder and activator transgenes. Mice expressing a transgene (CKII-tTA) [38] encoding a tetracycline-controlled transactivator under control of the calcium-calmodulin-dependent kinase IIα promoter were derived from mice that were a generous gift from Dr. Eric Kandel at Columbia University, New York, NY. These mice were successively backcrossed at least five times onto a 129S6 background strain. To construct the responder APP transgene, APP_NL_695 flanked by *Sal*I linkers was cloned into the unique *Xho*I site of MoPrP.Xho to generate prnp.APP_NL_. Next, the *Xba*I fragment of prnp.APP_NL_, including partial sequences of prnp introns 1 and 2, along with exons 2–3, and the APP_NL_ open reading frame, was cloned into the unique *Xba*I site in the inducible expression vector pTRE (Clontech, Inc., Palo Alto, CA), resulting in the plasmid pTRE.prnp.APP_NL_. The *London* mutation (V717I) was introduced into the pTRE.prnp.APP_NL_ plasmid using a site-directed mutagenesis kit (Stratagene, Santa Clara, CA), using the following primers: 5’-GCG ACA GTG ATC ATC ATC ACC TTG GTG ATG CTG-3’ and 5’-CAG CAT CAC CAA GGT GAT GAT GAT CAC TGT CGC-3’. The resulting plasmid (pTRE.prnp.APP_NLI_) was transformed into XL-1 blue competent cells (Stratagene) thate were plated on Lucia Broth plates with ampicillin. Clones were selected and plasmid DNA was purified using a miniprep kit (Qiagen, Valencia, CA) and sequenced for accuracy. The pTRE.prnp.APP_NLI_ plasmid was digested using *Asn*I, fractionated, and purified using a gel extraction kit (Qiagen) and dialysis. The concentration of the purified fragment containing the modified APP transgene was adjusted to 2 μg/mL, and was introduced by microinjection into the pronuclei of donor FVB/N embryos using standard techniques. Mice carrying the responder APP transgene (APP_NLI_) were maintained in the FVB/N strain, and identified by polymerase chain reaction (PCR) using the following primers: 5’-AAG CGG CCA AAG CCT GGA GGG TGG AAC A-3’ and 5’-GTT GAG CCT GTT GAT GCC CG-3’. The APP_NLI_ responders were subsequently mated to the activator TgCKII-tTA line, and pups positive for both transgenes were screened by PCR using the primer pairs 5’-GAT TAA CAG CGC ATT AGA GCT G-3’ and 5’-GCA TAT GAT CAA TTC AAG GCC GAT AAG-3’ for the activator transgene, and 5’-AAG CGG CCA AAG CCT GGA GGG TGG AAC A-3’ and 5’-GTT GAG CCT GTT GAT GCC CG-3’ for the responder transgene.

### Animals

All studies involving mice were conducted in full accordance with the guidelines of the Association for Assessment and Accreditation of Laboratory Animal Care (AAALAC) and approved by the Institutional Animal Care and Use Committee (IACUC) at the University of Minnesota (approval # 1202A09927). Animals were conventionally housed in plastic boxes with contact bedding and Nestlets. Mice were group-housed (maximum 4 per cage), except in the case of aggressive males, who were singly-housed. Animals were maintained on a 12-hour ON: 12-hour OFF light cycle, given *ad libitum* access to food and water, and monitored daily for evidence of injury or overtly aggressive behavior. For the study of transgene suppression, mice were administered doxycycline (200 ppm of chow) in their chow. At the ages enumerated below, mice were deeply anesthetized with isoflurane (absence of toe-pinch and corneal-blink reflexes) and decapitated for harvest of brains. All efforts were made to minimize suffering. Litters were sacrificed at pre-determined ages, and mice from each litter were randomly allocated to the different biochemical and histological studies.

### Human tissue

De-identified brain tissue samples were obtained from 6 elderly individuals enrolled in the Religious Orders Study, which was approved by the Institutional Review Board of Rush University Medical Center, Chicago, IL. Participants enroll without dementia and consent to annual clinical evaluation and sign an Anatomical Gift Act for organ donation. (Regarding this particular issue, please also see the following statement from Dr. David A. Bennett, principal investigator of the Religious Orders Study and director of the Rush Alzheimer's Disease Center—“Participants enroll without dementia and consent to annual clinical evaluation and sign an Anatomical Gift Act for organ donation. As this is a study until death, they are asked if they wish to designate someone with the power to remove them from the study at a later date should they become decisionally compromised in the future.”) These individuals were clinically and pathologically diagnosed with Alzheimer’s disease later. Proteins were extracted from the inferior temporal gyrus (Brodmann Area 20) using an adapted protocol of Shankar et al. [14]. Protein extraction and biochemical analyses were performed as described below.

As stated above, these samples were procured from ROS at the Rush Alzheimer's Disease Center, Chicago, Illinois. URL of the Rush Alzheimer's Disease Center— http://www.rush.edu/. URL of the ROS at the Rush Alzheimer's Disease Center— http://www.rush.edu/services-treatments/alzheimers-disease-center/religious-orders-study.

The specific samples used in this study have been described in a previous publication—Lesne SE, Sherman MA, Grant M, Kuskowski M, Schneider JA, et al. (2013) Brain amyloid-beta oligomers in ageing and Alzheimer's disease. Brain 136: 1383–1398.

### Preparation of synthetic Aβ oligomers and fibrils

A11-immunoreactive Aβ oligomers were prepared *in vitro* as previously described [19]. Briefly, 50 μg of Aβ was dissolved in 20 μL of hexafluoroisopropanol (HFIP) for 15 min at room temperature. The resulting Aβ solution was added to 180 μL of ddH_2_O in a siliconized Eppendorf tube. After 15 min incubation at room temperature, the samples were centrifuged for 15 min at 14,000 *g* and the supernatant fraction (pH 2.8–3.5) was transferred to a new siliconized tube, and the HFIP was evaporated off. The samples were then stirred using a Teflon coated micro stir bar for 24–48 hr at 22°C. OC-immunoreactive, soluble Aβ fibrillar aggregates were a kind gift from Dr. Robert Tycko, National Institues of Health, Bethesda, MD.

### Protein extraction

To better characterize protein of interest, we used three different extraction protocols to isolate proteins according to their solubility (three-step and two-step) and cellular compartmentalization (four-step).

A three-step protocol [14] was used to extract brain proteins that were used: 1) to measure levels of total Aβ in rTg9191 mice (Fig 5), 2) to measure levels of water-soluble Aβ dimers in rTg9191 mice (Fig 6A and 6B), and 3) to quantify soluble oligomers that are immunoreactive to OC or A11 antibodies in rTg9191 and Tg2576 mice and AD patients (Fig 7).

To prepare the water-soluble fraction, tissue specimens were weighed, transferred to 4 volumes (1 gm per 4 mL) of ice-cold buffer A (25 mM Tris-HCl, pH 7.4; 140 mM NaCl; 3 mM KCl; 0.1 mM phenylmethylsulfonyl fluoride; 0.2 mM 1,10-phenanthroline monohydrate; protease inhibitor cocktail (P8340, Sigma-Aldrich, St. Louis, MO); and phosphatase inhibitor cocktails (P2850 and P5726, Sigma-Aldrich)) and homogenized using a Dounce homogenizer. The resulting material was centrifuged for 90 min (16,100 *g*; 4°C); the supernatant was then depleted of endogenous immunoglobulins [42] and stored at -20°C until further use. The pellets were reserved for the extraction of detergent-soluble proteins.

To extract detergent-soluble proteins, the pellets obtained above were transferred to 4 volumes of ice-cold buffer B (25 mM Tris-HCl, pH 7.4; 140 mM NaCl; 3 mM KCl; 1% (v/v) Triton-X-100; 0.1 mM phenylmethylsulfonyl fluoride; 0.2 mM 1,10-phenanthroline monohydrate; protease inhibitor cocktail (P8340, Sigma-Aldrich); and phosphatase inhibitor cocktails (P2850 and P5726, Sigma-Aldrich)) and homogenized using a Dounce homogenizer. The resulting material was centrifuged for 90 min (16,100 *g*; 4°C); the supernatant was depleted of endogenous immunoglobulins and stored at -20°C until further use. The pellets were reserved for the extraction of detergent-insoluble proteins.

To extract insoluble proteins, the pellets obtained above were transferred to 40 μL of 70% formic acid and homogenized by repeatedly pipetting and vigorously shaking at room temperature for 30 min. The acidic pH of the resulting material was neutralized using 800 μL of 1 M Tris-base solution (no pH adjustment, pH ~10.6) and centrifuged for 90 min (16,100 *g*; 4°C). The supernatant was collected and stored at -20°C until further use.

A four-step fractionation protocol [15, 43] was employed 1) to determine the expression level of human APP (Fig 2B and 2C), 2) to determine the degree of DOX-mediated suppression of APP expression (Fig 2D and 2E), and 3) to investigate the regional pattern of APP expression (Fig 3).

Each hemi-forebrain was mechanically homogenized in 500 μL of Extraction Buffer 1 (50 mM Tris-HCl, pH 7.6; 150 mM NaCl; 2 mM EDTA; 0.1% (w/v) SDS; and 0.01% (v/v) NP-40, with the protease and phosphatase inhibitors mentioned above). Supernatants were collected after centrifugation (800 *g*; 10 min, 4°C) to obtain soluble, extracellular-enriched proteins. The resulting pellets were homogenized in 500 μL Extraction Buffer 2 (50 mM Tris-HCl, pH 7.6; 150 mM NaCl; and 0.1% (v/v) Triton-X-100, with protease and phosphatase inhibitors) followed by centrifugation (16,100 *g*; 90 min, 4°C). Supernatants were collected to obtain cytoplasmic proteins. The resulting pellets were gently agitated (15 min, 4°C) in 1 mL Extraction Buffer 3 (50 mM Tris-HCl, pH 7.4; 150 mM NaCl; 1 mM EGTA; 3% (w/v) SDS; 1% (w/v) deoxycholate; and 0.5% (v/v) Triton-X-100, and protease and phosphatase inhibitors); subsequent centrifugation (16,100 *g*; 90 min, 4°C) yielded a supernatant enriched in membrane-associated proteins and a pellet containing detergent-insoluble proteins. The three detergent-soluble fractions were then depleted of endogenous immunoglobulins. Detergent-insoluble pellets were incubated with 40 μL of 70% formic acid, mechanically dissociated, gently agitated, and neutralized with 800 μL of 1 M Tris-base solution. Samples were centrifuged (16,100 *g*; 90 min, 4°C) and the supernatants were collected and concentrated to ~350–400 μL/sample using a vacuum concentrator (Eppendorf Vacufuge 5301, room temperature, ~2 hr). Protein extracts were stored at -20°C until further use.

Finally, material obtained from a two-step brain protein extraction protocol [14] was used 1) to measure levels of Aβ*56 in rTg9191, TgArc6, and hAPP-J20 mice (Fig 6C and 6D), and 2) to determine levels of CTFβ in rTg9191 and Tg2576 mice (S1 Fig). Forebrains were mechanically homogenized in 500 μL of extraction buffer (50 mM Tris-HCl, pH 7.4; 150 mM NaCl; 1 mM EGTA; 3% (w/v) SDS; 0.5% (v/v) Triton-X-100; 1% (w/v) deoxycholate; and protease and phosphatase inhibitors described above). Homogenates were gently agitated at 4°C for 1 hr, and then centrifuged for 90 min (16,100 *g*; 4°C). The resulting supernatant was depleted of endogenous immunoglobulins and stored at -20°C until further use. Pellets were extracted using 40 μL of 70% formic acid and then neutralized with 400 μL of 1 M Tris-base solution. Samples were then centrifuged (16,100 *g*; 90 min, 4°C); supernatants were collected and stored at -20°C until further use.

Protein concentrations of brain extracts were determined using a BCA protein assay kit (23225, Thermo Scientific, Rockford, IL) according to the manufacturer’s instructions.

### Immunoblot

#### Western blotting

Western blotting was performed using the protocol of Liu et al. [42].

Proteins were denatured by heating under reducing conditions. Tricine loading buffer (450 mM Tris-HCl, pH 8.0; 24% (v/v) glycerol; 8% (w/v) SDS; 0.01% (w/v) Coomassie Brilliant Blue; 0.1% (v/v) Phenol Red; 5% (v/v) β-mercaptoethanol) was added to each sample (1:3, v/v, buffer:sample), and the samples were heated with agitation at 95°C for 5 min. The denatured samples were loaded onto pre-cast 10–20% SDS-polyacrylamide Tris-Tricine gels (345–0067, Bio-Rad, Hercules, CA) and electrophoretically separated at room temperature at constant voltage (80 V).

The separated proteins were then transferred to nitrocellulose membranes (162–0112, Bio-Rad) under low temperature conditions (transfer performed at 4°C, 0.4 A constant current, 4 hr). Membranes were then transferred to 50 mL of PBS (prepared by dissoving one pellet of phosphate buffered saline (P4417, Sigma-Aldrich) into 200 mL of ddH_2_O), and epitopes were retrieved by boiling the membranes twice for 10 sec each time, with a 4 min cooling period after each episode of boiling. Non-specific binding sites were blocked by incubating the membranes on an orbital shaker for 1–3.5 hr at room temperature in blocking buffer (10 mM Tris-HCl, pH 7.4; 200 mM NaCl; 0.1% (v/v) Tween-20; 33% (v/v) casein blocking buffer (C7594, Sigma-Aldrich); 0.05% (w/v) cold water fish skin gelatin (G7041, Sigma-Aldrich); 5% (w/v) bovine serum albumin (BSA) (A3803, Sigma-Aldrich)). Primary antibodies were then added directly to the blocking buffer as follows: mouse monoclonal 22C11 (MAB348, Millipore, Billerica, MA; directed against amino acids 66–81 of APP), 1:2,000; mouse monoclonal 4G8 (SIG-39220, Covance, Princeton, NJ; directed against amino acids 17–24 of Aβ), 1:10,000; mouse monoclonal 6E10 (SIG-39320, Covance; directed against amino acids 1–16 of human Aβ), 1:2,500; biotinylated 6E10 (SIG-39340, Covance), 1:2,500; mouse monoclonal LN27 (SIG-39188, Covance; directed against an epitope within the first 200 amino acids of human APP), 1:2,500; mouse monoclonal anti-α-tubulin (T5168, Sigma-Aldrich), 1:200,000. Membranes were incubated overnight at 4°C on an orbital shaker. Following primary antibody incubation, membranes were rinsed once briefly with TBST wash buffer (10 mM Tris-HCl, pH 7.4; 200 mM NaCl; 0.1% (v/v) Tween-20) at room temperature, and then an additional five times for 5 min each on the orbital shaker. Membranes were then incubated for 1 hr at room temperature with secondary antibody (for Figs 2B, 2D, 3 and 6A lower panel and 6C lower panel, horseradish peroxidase (HRP)-conjugated goat-anti-mouse IgG (31437, Thermo Scientific), diluted 1:200,000 in TBST wash buffer; for Fig 6A upper panel, biotin-conjugated goat-anti-mouse IgG (31805, Thermo Scientific), diluted 1: 60,000 in blocking buffer; gently agitated on the orbital shaker). When biotin-conjugated 6E10 was used (Fig 6C upper panel), secondary antibody incubation was skipped. Following secondary-antibody incubation, membranes were washed in TBST as after primary-antibody incubation. Membranes that had been incubated with biotin-conjugated secondary antibodies were then incubated at room temperature with HRP-conjugated NeutrAvidin (A2664, Invitrogen, Carlsbad, CA; diluted 1:5,000 in TBST wash buffer) for 7–8 min, with agitation (orbital shaker), and then rinsed with TBST as above. These membranes, as well as the membranes that had been incubated with HRP-conjugated secondary antibodies, were then developed using a chemiluminescence reagent (SuperSignal West Pico Chemiluminescent Substrate, 34080, Thermo Scientific; 4 min, room temperature, orbital shaker). Chemiluminescence was detected using Kodak Scientific Imaging film X-OMAT Blue XB (1776699, Perkin-Elmer Life Sciences Inc., Boston, MA) processed in a Konica medical film processor (Model SRX-101A, Konica Medical Imaging Inc., Wayne, NJ). For each blot, a series of exposures, ranging from 1 sec to 5 min, was used to ensure that bands of interest fell within the linear range of detection. Intensities of immunoreactive bands were quantified densitometrically using Optiquant (Packard Cyclone, Perkin-Elmer Life Sciences Inc.).

After being probed with anti-APP or anti-Aβ antibodies, the same blots were chemically stripped using Restore PLUS Western Blot Stripping Buffer (46430, Thermo Scientific) at room temperature for 1 hr. The membranes were then re-probed with anti-α-tubulin to examine the levels of total protein loaded onto the membrane according to the protocol described above.

The numbers of samples used were: 1) to determine the expression level of transgenic APP (Fig 2B and 2C): rTg9191 mice at 2 months of age, n = 3 (1 male and 2 females); age-matched non-transgenic littermates, n = 3 (1 male and 2 females); 2) to determine the degree of DOX-mediated suppression of the APP transgene (Fig 2D and 2E): rTg9191 mice at 8 months of age, n = 3 (2 males and 1 female); at 10 months of age, n = 3 (2 males and 1 female); at 10 months of age, with DOX treatment between 8–10 months of age, n = 3 (2 males and 1 female); non-transgenic littermates at 8 months of age, n = 3 (2 males and 1 female); 3) to investigate the regional expression pattern of transgenic APP (Fig 3): rTg9191 mice at 12 months of age, n = 2 (1 male and 1 female); age-matched non-transgenic littermates, n = 2 (1 male and 1 female); 4) to measure levels of Aβ dimers (Fig 6A and 6B): rTg9191 mice at 4 months of age, n = 4 (2 males and 2 females); at 12 months of age, n = 5 (2 males and 3 females); at 21 months of age, n = 3 (1 male and 2 females); at 24 months of age, n = 3 (1 male and 2 females); at 26 months of age, n = 6 (3 males and 3 females); non-transgenic littermates at 26 months of age, n = 3 (1 male and 2 females); and 5) to measure levels of Aβ*56 (Fig 6C and 6D): rTg9191 mice at 4 months of age, n = 3 (1 male and 2 females); at 12 months of age, n = 5 (2 males and 3 females); at 21 months of age, n = 5 (2 males and 3 females); at 24 months of age, n = 6 (3 males and 3 females); at 26 months of age, n = 8 (4 males and 4 females); non-transgenic littermates at 26 months of age, n = 3 (1 male and 2 females); TgArc6 mice at 4 months of age, n = 5 (2 males and 3 females); hAPP-J20 mice at 4 months of age, n = 5 (2 males and 3 females). Each experiment was repeated three times.

#### Dot blotting

For dot blots probed with OC antibodies, 0.5 μg of water-soluble protein from brain extracts and 2 ng of synthetic soluble Aβ fibrillar aggregates were spotted onto nitrocellulose membranes. Membranes were first rinsed three times with Tris-buffered saline (TBS) at room temperature for 5 min each, and then incubated in 10% (w/v) non-fat milk in Tris-buffered saline with 0.01% (v/v) Tween-20 (TBS-T) at room temperature for 1 hr. Membranes were then washed five times with TBS-T for 4 min each on an orbital shaker, and then incubated overnight with OC antibodies (AB2286, Millipore; diluted 1:50,000 in TBS-T with 5% (w/v) BSA) at 4°C. Membranes were then washed five times with TBS-T at room temperature for 5 min each, and then incubated for 1 hr with secondary antibody solution at room temperature HRP-conjugated goat-anti-rabbit IgG (31463, Thermo Scientific; diluted 1:200,000 in TBS-T).

A similar protocol was applied to dot blots probed with A11 antibodies except that: 1) 1 μg of water-soluble protein extracts and 1 μg of synthetic Aβ oligomers were dotted, 2) membranes were blocked with 5% (w/v) BSA in TBS, 3) A11 antibodies (a kind gift from Dr. Rakez Kayed, University of Texas, Galveston, TX) were diluted 1:2,000 in blocking buffer, 4) membranes were washed with TBS, and 5) secondary antibody was applied to membranes in TBS.

To confirm that the immunoreactivities of conformation-selective antibodies came from Aβ assemblies, we performed immunodepletion: Brain extracts and synthetic Aβ aggregates were also incubated overnight with a combination of four anti-Aβ antibodies (i.e., 6E10, 4G8; and rabbit monoclonals 139–5 (SIG-39166, Covance) and 1-11-3 (SIG-39169, Covance)—two C-terminal end-specific antibodies directed against Aβ40 and Aβ42, respectively) at 4°C. Antibody-Aβ complexes and any remaining free antibodies were removed by three rounds of precipitation using Protein G Sepharose 4 FF resin (17-0618-01, GE Healthcare, Piscataway, NJ). Immunodepleted materials of the same portion as the original samples were dotted onto the same membrane to make a direct comparison to the untreated samples.

Blot development and densitometry-based quantitative analysis were similarly performed as described in **Western blotting**.

After being probed with conformation-sensitive antibodies, the same blots were chemically stripped using Restore PLUS Western Blot Stripping Buffer at room temperature for 1 hr. The membranes were then re-probed with anti-α-tubulin, according to the standard Western blot protocol described above, to confirm equal loading of protein across samples.

The numbers of samples examined in dot blots are: rTg9191 mice at 4 months of age, n = 3 (1 male and 2 females); at 12 months of age, n = 3 (1 male and 2 females); at 21 months of age, n = 3 (1 male and 2 females); at 24 months of age, n = 3 (1 male and 2 females); non-transgenic littermates of rTg9191 mice at 4 months of age, n = 3 (1 male and 2 females); at 24-months of age, n = 3 (1 male and 2 females); Tg2576 mice at 21 months of age, n = 3 (1 male and 2 females); age-matched non-transgenic littermates of Tg2576, n = 3 (1 male and 2 females); AD patients, n = 3 (2 males and 1 female). Each experiment was repeated three times.

### Enzyme-linked immunosorbent assay (ELISA)

Total Aβ38, Aβ40, and Aβ42 protein levels in the water-soluble (TBS), detergent-soluble (TBS-TX) and insoluble (FA) fractions of brain extracts of rTg9191 mice at young (4-month-old, n = 3 (1 male and 2 females)), middle (12-month-old, n = 5 (2 males and 3 females)), and old (21-month-old, n = 3 (1 male and 2 females); and 24-month-old, n = 5 (2 males and 3 females)) ages were measured using a multi-plex ELISA for Aβ38, Aβ40, and Aβ42 (N45148A-1, Meso Scale Discovery, Rockville, MD) following the manufacturer’s instructions. In this assay, C-terminal end-specific antibodies against Aβ38, Aβ40, and Aβ42 are used as immunocapture reagents, and mouse monoclonal antibody 6E10 directed against amino acids 1–16 of human Aβ is used as the detection antibody. Signals were detected and quantified using a Meso Sector S 600 plate reader (MesoScale Discovery). Samples were analyzed in duplicate, and the mean values are reported.

### Histology and immunohistochemistry

The rTg9191 mice were aged and sacrificed at two-month intervals from 2–26 months of age. Mice were weighed immediately before they were anesthetized using isoflurane and killed by decapitation. After mice were sacrificed, brains were immediately dissected, and weights of whole brains and right forebrains were recorded. Left hemispheres were immersion-fixed in 10% formalin for 24–48 hr, then embedded in paraffin. Brains were serially sectioned in the parasagittal plane using a Leica RM2255—Fully Motorized Rotary Microtome (Leica Microsystems, Buffalo Grove, IL) and mounted onto CapGap slides (Fisher Scientific, Pittsburgh, PA).

#### Amyloid plaques

To investigate the age-dependent progression of Aβ plaques, eight transgenic mice (APP/TTA) (four males and four females), two littermates expressing only the TTA activator (TTA) (one male and one female), two harboring only the APP responder gene (APP) (one male and one female) and two non-transgenics (non-Tg) (one male and one female) were analyzed. Three 5 μm-thick parasagittal sections from each animal, at ~0.84, 1.20, and 1.56 mm lateral from the midline [44], were prepared. Selected sections were deparaffinized and rehydrated according to standard protocols. For epitope retrieval, mounted slides were pretreated in 70% formic acid at room temperature for 5 min. Tissue sections were subsequently blocked with Background Sniper (Universal) blocking reagent (BS966 MM, Biocare Medical, Concord, CA) at room temperature for 1 hr, then incubated with monoclonal antibodies 6E10 (1:8,000), 4G8 (1:3,000), 139–5 (1:500), and 1-11-3 (1:400) at 4°C overnight. Aβ immunostained profiles were visualized using diaminobenzidine chromagen. Hematoxylin and eosin counterstaining was used to provide cytological detail.

#### Quantification of amyloid burden

Amyloid plaques, immunostained with 4G8, were viewed with an Axio Imager upright microscope (Carl Zeiss Microimaging GmbH, Göettingen, Germany) equipped with an AxioCam MRc color digital camera. Stereology-based quantification of amyloid burden was carried out using Stereo Investigator 9 software (MBF Bioscience, Chicago, IL). The entire cerebral cortex and hippocampus were separately sampled with the counting frame size 250 μm × 250 μm for cortex and 100 μm × 100 μm for hippocampus. The sum of the area of all amyloid plaques was divided by the total area of cerebral cortex or hippocampus to obtain the amyloid burden. Experimenters performing quantification of amyloid burden were blind to age and genotype of mice.

#### Glial markers and hyperphosphorylated tau

To investigate gliosis and tau pathology, five rTg9191 mice (24 months of age, 2 males and 3 females), four age-matched non-transgenic littermates (2 males and 2 females), two age-matched non-transgenic littermates expressing only TTA (1 male and 1 female), three Tg2576 (23 months of age, 1 male and 2 females) mice, and one rTg4510 (15 months of age, female) mouse were used. Formalin-fixed, paraffin-embedded (FFPE) hemispheres were sectioned at 10 μm in the parasagittal plane. Sections were de-paraffinized and rehydrated using standard methods, then incubated in 1× Reveal Decloaker buffer (RV 1000 M, Biocare Medical) in a steamer for 30 min at 95–98°C for antigen retrieval. Endogenous peroxidase activity was quenched in 3% (v/v) hydrogen peroxide solution (Peroxidized, Biocare Medical) for 10 min. Tissue sections were subsequently blocked with serum-free blocking solution (Rodent Block M (RBM961 MM, Biocare Medical)) at room temperature for 15 min. Blocking solution was removed and slides were incubated in primary antibodies diluted in 10% blocking solution/90% TBS (v/v) for 1 hr at room temperature. The following primary antibodies were used: 1) to probe astrocytes and microglial cells: rabbit polyclonals anti-S100 (1:40,000) (Z0311, DAKO, Carpinteria, CA), anti-GFAP (1:30,000) (Z0334, DAKO) and anti-Iba1 (1:2,000) (019–19741, Wako Chemicals, Richmond, VA); and 2) to probe hyperphosphorylated and misfolded tau: mouse monoclonals AT8 (1:400) (MN1020B, Thermo Scientific), Alz50 (1:100), CP13 (1:4,000), MC1 (1:800), PG5 (1:200), PHF-1 (1:1,500) and TG-3 (1:100) (the latter 6 antibodies are kind gifts from Dr. P. Davies, Albert Einstein College of Medicine, New York, NY). Immunoreactivities were visualized using diaminobenzidine chromagen. The sections were then counterstained in 1% (w/v) Congo red (C6277, Sigma-Aldrich) aqueous solution for 1 hr at room temperature to visualize dense-core plaques. Finally, hematoxylin and eosin staining was used to provide cytological detail.

#### Axonal pathology

To study dense-core plaques and axonal pathology, FFPE hemispheres from three 24-month-old rTg9191 mice (1 male and 2 females) and two non-transgenic littermates (1 male and 1 female) were sectioned at 16 μm in the parasagittal plane. To visualize axon structure, every 15^th^ section (240 μm interval) was rehydrated; subjected to antigen retrieval (treated with 1× Reveal Decloaker buffer in a Black & Decker steamer for 35 min with a 20-min cool down); blocked (Rodent Block M, room temperature for 1 hr); then incubated with monoclonal antibody SMI-312 (SMI-312R, Covance), directed against axonal neurofilaments (diluted 1:2,000 in 10% (v/v) Background Sniper (Universal) blocking reagent, 4°C, overnight) followed by Alexa Fluor 555-conjugated donkey-anti-mouse (A31570, Invitrogen; diluted 1:2,000 in 10% Background Sniper (Universal) blocking reagent). To reveal dense-core plaques, sections immunostained with SMI-312 were counterstained with freshly prepared 1% (w/v) thioflavin-S (T1892, Sigma-Aldrich) aqueous solution at room temperature for 5 min followed by differentiation using 70% ethanol.

For all immunostaining, sections were also exposed to secondary antibodies only. In such cases, no positive labeling was observed.

#### Hematoxylin and eosin stain

To visualize brain structure, eight APP/TTA (four males and four females), two TTA (one male and one female), two APP (one male and one female) and two non-Tg (one male and one female) mice were analyzed. Three 5 μm-thick sections from each animal, at ~0.84, 1.20, and 1.56 mm lateral from midline, were prepared. Hematoxylin and eosin staining was performed on selected sections according to standard protocols.

### Statistics

Statistics were performed using StatView Version 5.0.1 (SAS Institute Inc., Cary, NC). Data are expressed as mean ± SEM.

## Supporting Information

S1 Fig Beta-secretase-mediated APP processing in rTg9191 mice. (A) Representative blots show levels of C-terminal fragment (CTFβ) generated by β-secretase cleavage of APP. The production of CTFβ was studied at four ages (12, 21, 24 and 26 months); in addition, levels of CTFβ from mice (26M*) treated with DOX for 2 months starting at 24 months of age were also examined. Membrane-enriched fraction of brain extracts was immucaptured with 6E10; CTFβ (upper panels) and full-length APP (fl-APP) (middle panels, short exposure) were revealed by anti-APP antibodies (directed against an epitope within C-terminus of APP). Immunoblots of immunoglobulin heavy chain(IgG_H_) (lower panels) show that equal amounts of capture antibody were used to react with each sample. For each blot, two 20 month-old Tg2576 mice were used as internal controls for comparing levels of proteins between different blots. No Ab.: no capture antibody was included in immunoreactions; No. Extr.: no protein extracts were included in immunoreactions; No Extr. or Ab.: only matrix was included. Asterisk (*) between fl-APP and CTFβ in upper panels: non-specific signals. (B-C) Quantification of fl-APP (B) and CTFβ (normalized to fl-APP) (C) of rTg9191 and Tg2576 mice. Genders of mice whose brain extracts were used in the Western blots, aligning in the order of left to right, are: N/A, F(24-month-old), N/A, M, F, M, F, F, F, M, F, F; M, F, F, M, F, M, F, F, F, M, M, F; M, F, M, F, M, F, F, F, M, F, M, F; M, F, M, F, M, F, F, F, M, M, M, F (N/A, no extracts applied; M, male; F, female).
(TIF)

S2 Fig Amyloid plaque pathology of rTg9191 mice. (A-D) Representative high magnification photomicrographs show details of dense-core (arrowheads) and diffuse (arrows) plaques recognized by 6E10 (A), 4G8 (B), 139–5 (anti-Aβ_x-40_) (C) and 1-11-3 (anti-Aβ_x-42_) (D). (E-F) Representative photomicrographs show dense-core plaques stained by thioflavin S in cerebral cortex (E) and hippocampus (F). Scale bars: 50 μm (A-D), 200 μm (E-F). All photomicrographs represent brain sections of female mice.
(TIF)

S3 Fig Neuroinflammation and abnormal neuronal architecture in rTg9191 mice and littermates expressing only tetracycline transactivator (TTA). (A-D) TTA mice showed no apparent reactive gliosis. Brain sections from TTA (A,C)and rTg9191 mice (B,D) at 24 months of age were stained with a monoclonal antibody directed against the microglial marker ionized calcium-binding adaptor molecule 1 (Iba1) (A,B), and an antibody directed against the astrocytic marker glial fibrillary acidic protein (GFAP) (C,D). Congo red was then applied to stain plaques. Representative photomicrographs show staining of the molecular layer of dentate gyrus. No plaques were detected in TTA mice and no activated microglial cells or astrocytes were overtly observed. Scale bars in B and D, 25 μm, applies to A-D. (E) TTA mice exhibited no dystrophic neurites. Brain sections were stained with monoclonal antibody SMI-312 to visualize axons (red) and counterstained using thioflavin S. Scale bar, 50 μm. Compare image in (E) to Fig 9J and 9K. All photomicrographs are of brain sections of female mice.
(TIF)

S4 Fig Tau pathology in rTg9191 and TTA mice. Brain sections of TTA (A,C,E,G,I,K,M) and rTg9191 mice (B,D,F,H,J,L,N) stained with antibodies directed against pathological conformation- and phosphorylation-dependent epitopes of tau: AT8 (A,B), CP13 (C,D), PG5 (E,F), PHF-1 (G,H), Alz50 (I,J), MC1 (K,L) and TG-3 (M,N) and counterstained with Congo red. Representative photomicrographs show staining of the molecular layer of the dentate gyrus. No hyperphosphorylated and/or misfolded tau was observed in TTA mice. Scale bars: 20 μm, applies to all images. All photomicrographs are of brain sections of female mice.
(TIF)

S1 Text Supplementary Materials and Methods. (DOC)
