# Supplementary figures and images for: Characterization of a Novel Mouse Model of Alzheimer’s Disease—Amyloid Pathology and Unique β-Amyloid Oligomer Profile
Source: PLoS One. 2015 May 6;10(5):e0126317. doi: 10.1371/journal.pone.0126317 (PMC4422728; doi:10.1371/journal.pone.0126317)

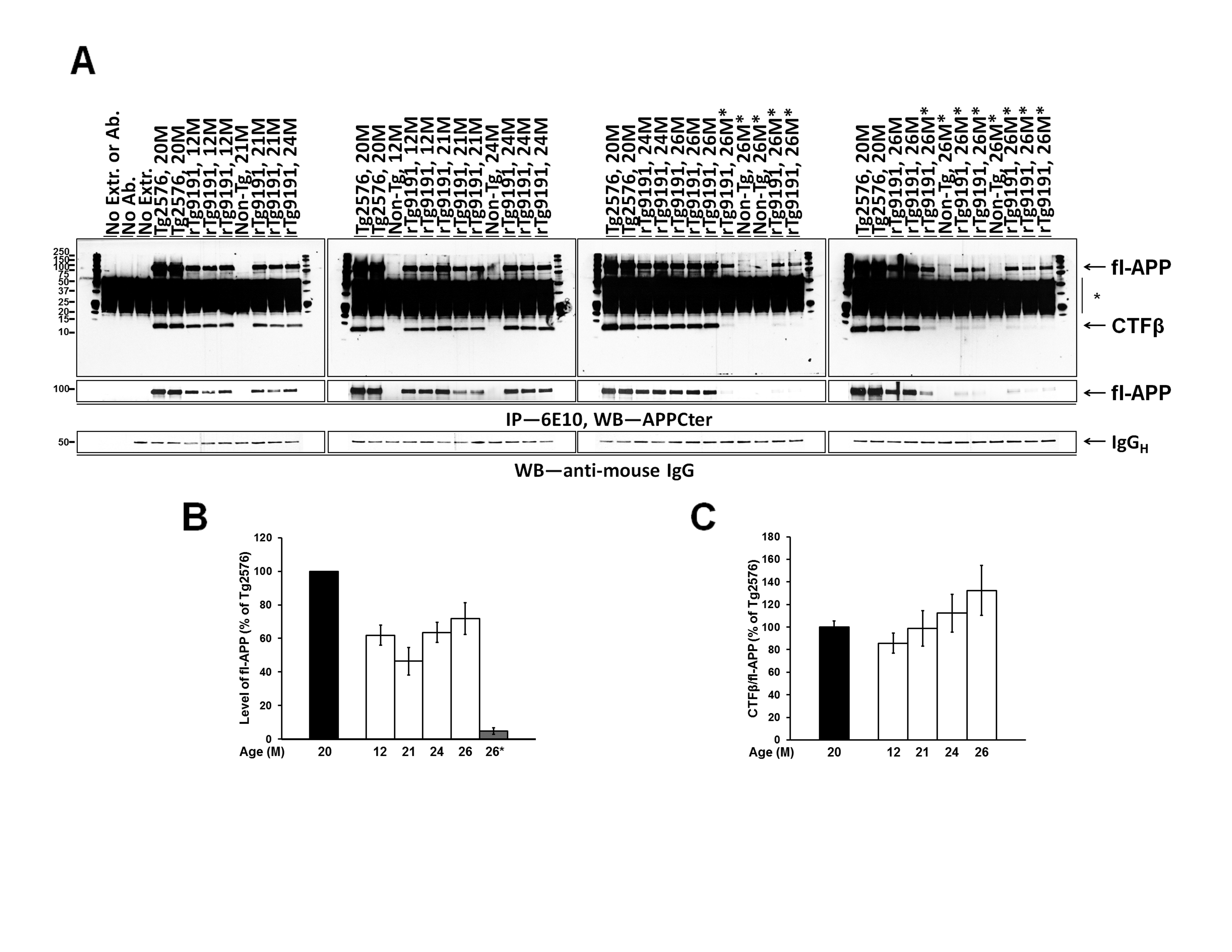

Supplement: S1 Fig — (A) Representative blots show levels of C-terminal fragment (CTFβ) generated by β-secretase cleavage of APP. The production of CTFβ was studied at four ages (12, 21, 24 and 26 months); in addition, levels of CTFβ from mice (26M*) treated with DOX for 2 months starting at 24 months of age were also examined. Membrane-enriched fraction of brain extracts was immucaptured with 6E10; CTFβ (upper panels) and full-length APP (fl-APP) (middle panels, short exposure) were revealed by anti-APP antibodies (directed against an epitope within C-terminus of APP). Immunoblots of immunoglobulin heavy chain(IgGH) (lower panels) show that equal amounts of capture antibody were used to react with each sample. For each blot, two 20 month-old Tg2576 mice were used as internal controls for comparing levels of proteins between different blots. No Ab.: no capture antibody was included in immunoreactions; No. Extr.: no protein extracts were included in immunoreactions; No Extr. or Ab.: only matrix was included. Asterisk (*) between fl-APP and CTFβ in upper panels: non-specific signals. (B-C) Quantification of fl-APP (B) and CTFβ (normalized to fl-APP) (C) of rTg9191 and Tg2576 mice. Genders of mice whose brain extracts were used in the Western blots, aligning in the order of left to right, are: N/A, F(24-month-old), N/A, M, F, M, F, F, F, M, F, F; M, F, F, M, F, M, F, F, F, M, M, F; M, F, M, F, M, F, F, F, M, F, M, F; M, F, M, F, M, F, F, F, M, M, M, F (N/A, no extracts applied; M, male; F, female). (TIF) [file pone.0126317.s001.tif]

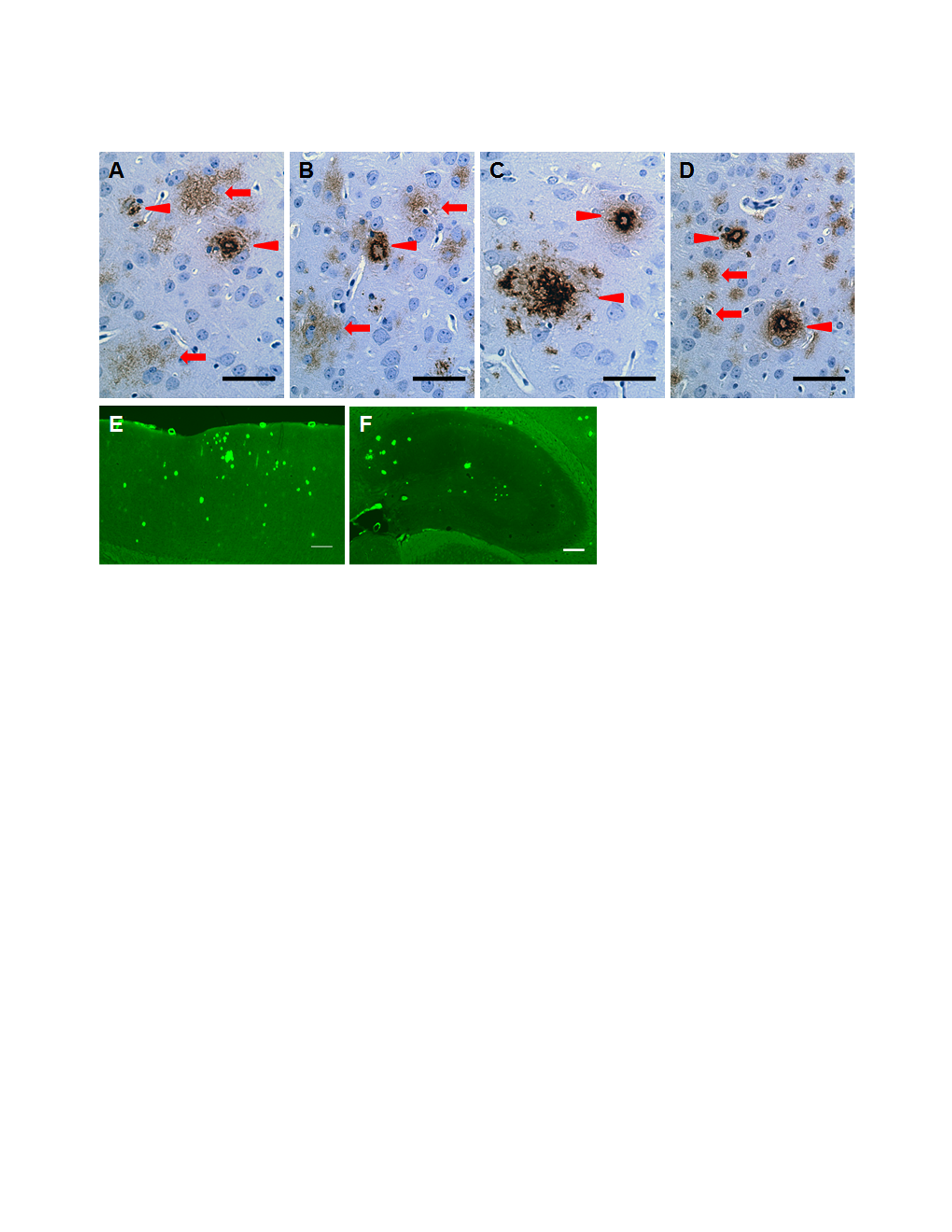

Supplement: S2 Fig — (A-D) Representative high magnification photomicrographs show details of dense-core (arrowheads) and diffuse (arrows) plaques recognized by 6E10 (A), 4G8 (B), 139–5 (anti-Aβx-40) (C) and 1-11-3 (anti-Aβx-42) (D). (E-F) Representative photomicrographs show dense-core plaques stained by thioflavin S in cerebral cortex (E) and hippocampus (F). Scale bars: 50 μm (A-D), 200 μm (E-F). All photomicrographs represent brain sections of female mice. (TIF) [file pone.0126317.s002.tif]

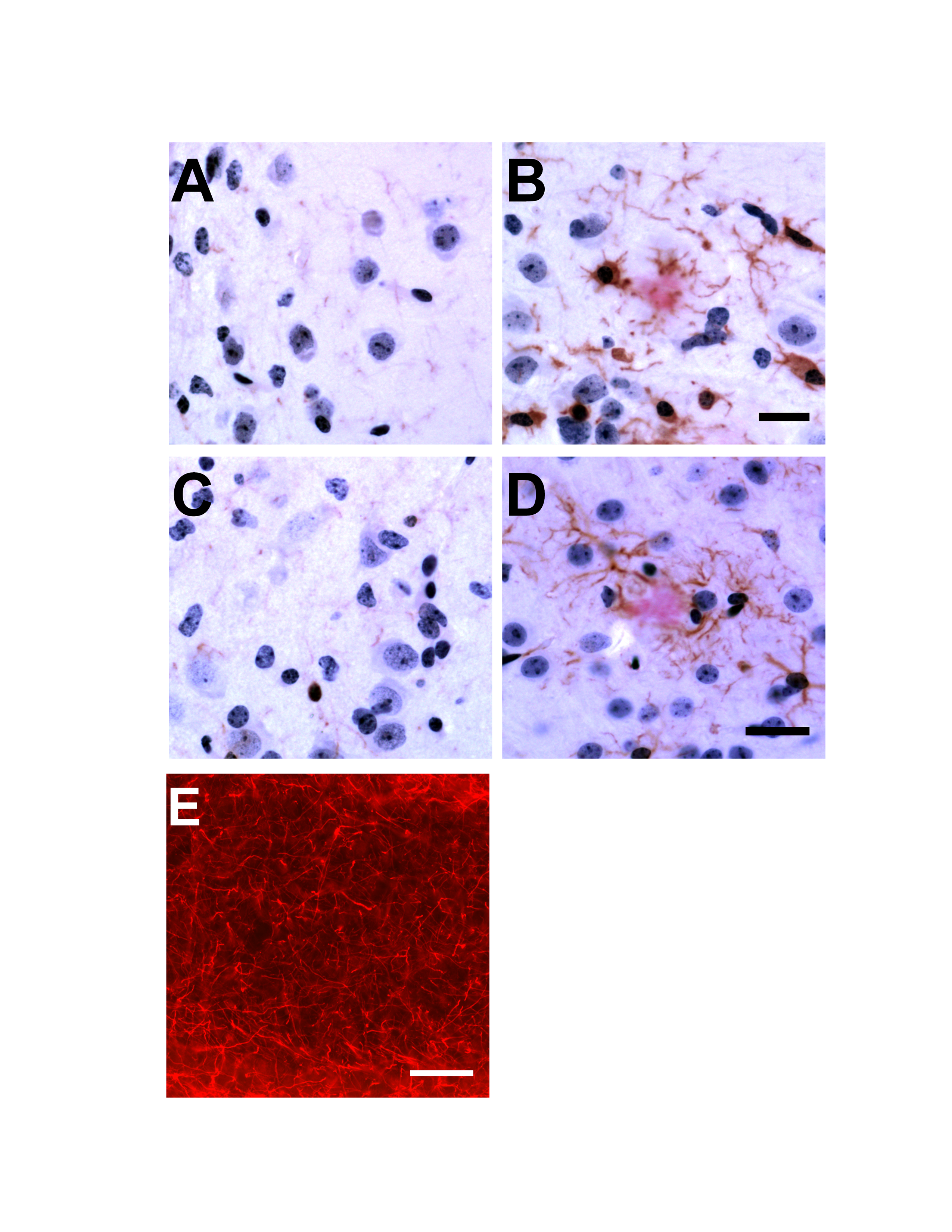

Supplement: S3 Fig — (A-D) TTA mice showed no apparent reactive gliosis. Brain sections from TTA (A,C)and rTg9191 mice (B,D) at 24 months of age were stained with a monoclonal antibody directed against the microglial marker ionized calcium-binding adaptor molecule 1 (Iba1) (A,B), and an antibody directed against the astrocytic marker glial fibrillary acidic protein (GFAP) (C,D). Congo red was then applied to stain plaques. Representative photomicrographs show staining of the molecular layer of dentate gyrus. No plaques were detected in TTA mice and no activated microglial cells or astrocytes were overtly observed. Scale bars in B and D, 25 μm, applies to A-D. (E) TTA mice exhibited no dystrophic neurites. Brain sections were stained with monoclonal antibody SMI-312 to visualize axons (red) and counterstained using thioflavin S. Scale bar, 50 μm. Compare image in (E) to Fig 9J and 9K. All photomicrographs are of brain sections of female mice. (TIF) [file pone.0126317.s003.tif]

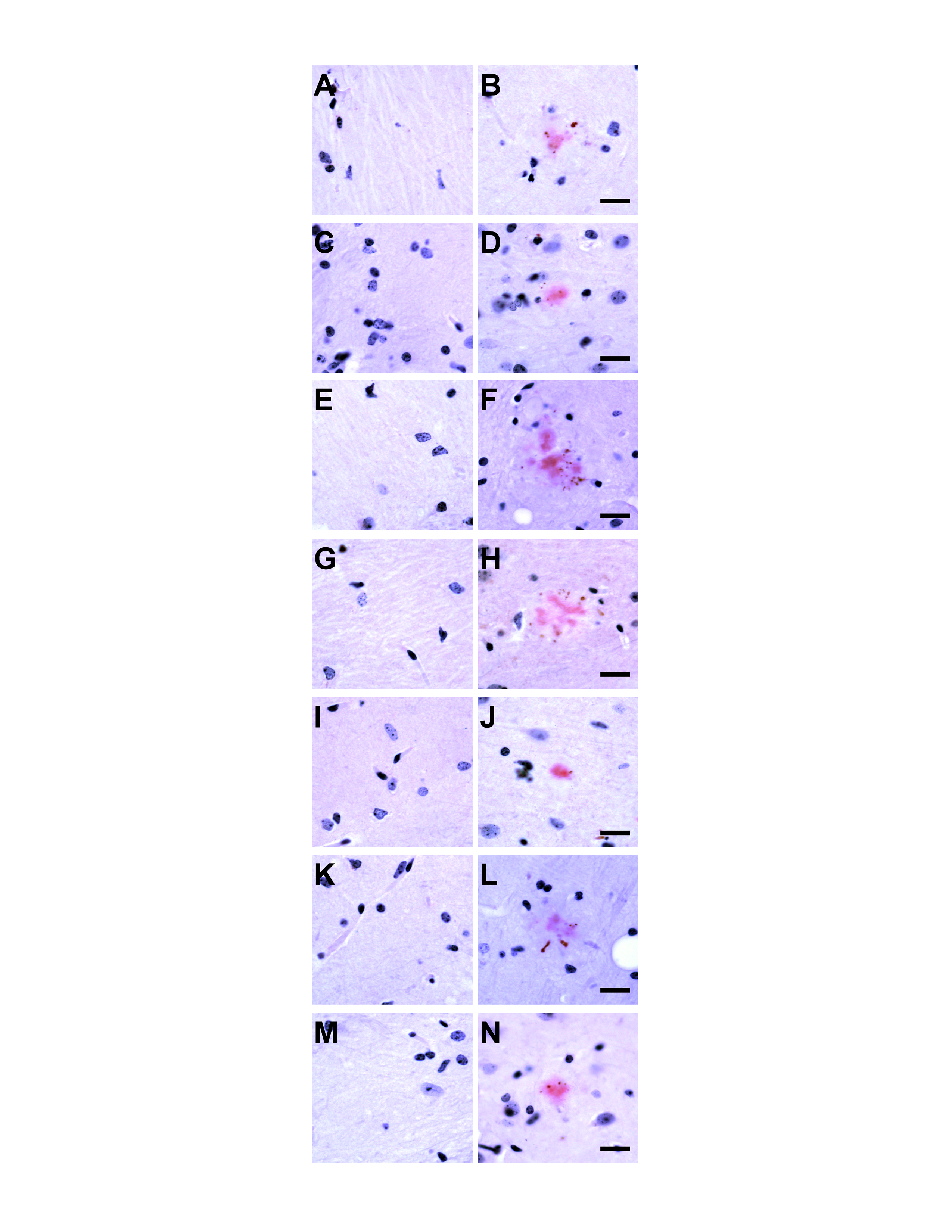

Supplement: S4 Fig — Brain sections of TTA (A,C,E,G,I,K,M) and rTg9191 mice (B,D,F,H,J,L,N) stained with antibodies directed against pathological conformation- and phosphorylation-dependent epitopes of tau: AT8 (A,B), CP13 (C,D), PG5 (E,F), PHF-1 (G,H), Alz50 (I,J), MC1 (K,L) and TG-3 (M,N) and counterstained with Congo red. Representative photomicrographs show staining of the molecular layer of the dentate gyrus. No hyperphosphorylated and/or misfolded tau was observed in TTA mice. Scale bars: 20 μm, applies to all images. All photomicrographs are of brain sections of female mice. (TIF) [file pone.0126317.s004.tif]
