# Supplementary material for: Characterization of a Novel Mouse Model of Alzheimer’s Disease—Amyloid Pathology and Unique β-Amyloid Oligomer Profile
Source: PLoS One. 2015 May 6;10(5):e0126317. doi: 10.1371/journal.pone.0126317 (PMC4422728; doi:10.1371/journal.pone.0126317)
Supplement: S1 Text — (DOC) [file pone.0126317.s005.doc]

Supporting Information *PLOS ONE*

**Characterization of a novel mouse model of Alzheimer’s disease**

**– amyloid pathology and unique Aβ oligomer profile**

**Peng Liu,^1,3,*^ Jennifer B. Paulson,^1,3^ Colleen L. Forster,^2,3^ Samantha L. Shapiro,^1,3^ Karen H. Ashe,^1,3,4,5^ Kathleen R. Zahs^1,3,*^**

^1^Department of Neurology, University of Minnesota, Minneapolis, Minnesota, United States of America

^2^University of Minnesota Academic Health Center Biological Materials Procurement Network (BioNet), University of Minnesota, Minneapolis, Minnesota, United States of America

^3^N. Bud Grossman Center for Memory Research and Care, University of Minnesota, Minneapolis, Minnesota, United States of America

^4^Department of Neuroscience, University of Minnesota, Minneapolis, Minnesota, United States of America

^5^Geriatric Research Education and Clinical Centers, Veterans Affairs Medical Center, Minneapolis, Minnesota, United States of America

*****Corresponding authors: [liuxx726@umn.edu](mailto:liuxx726@umn.edu) (PL), [zahsx001@umn.edu](mailto:zahsx001@umn.edu) (KRZ)

**Supplementary Materials and Methods**

*Immunoprecipitation (IP).* To determine levels of β-secretase-cleaved APP C-terminal fragments (CTFβ), 50 µg of detergent-soluble extracts were incubated with 4 μg of 6E10, and immuno-complexes were captured by incubation with 30 µL of nProtein A Sepharose 4 FF resin (17-5280-01, GE healthcare) at 4°C for 14 – 16 hr. The resulting resin was washed with buffer A (50 mM Tris-HCl, pH 7.4; 300 mM NaCl; 1 mM EDTA; 0.1% (v/v) Triton-X-100) once, followed by buffer B (50 mM Tris-HCl, pH 7.4; 150 mM NaCl; 1 mM EDTA; 0.1% (v/v) Triton-X-100) once at 4°C for 20 min each; 30 μL of loading buffer (500 mM Tris-HCl, pH 8.0; 24% (v/v) glycerol; 8% (w/v) sodium dodecyl sulfate (SDS); 0.01% (w/v) Coomassie Brilliant Blue; 0.1% (v/v) phenol red; 0.1% (v/v) β-mercaptoethanol) was added to the resin; heated at 96°C while shaking for 10 min, and spun at 9,300 *g* at room temperature for 5 min. The supernatant was resolved by SDS-PAGE, and full-length APP and CTFβ were revealed by rabbit polyclonal anti-APP C-terminal antibodies (36-6900, Invitrogen, 1: 10,000).

The numbers of samples used: rTg9191 mice at 12 months of age, n = 5 (2 males and 3 females); at 21 months of age, n = 5 (2 males and 3 females); at 24 months of age, n = 6 (3 males and 3 females); at 26 months of age with no DOX treatment, n = 5 (2 males and 3 females); at 26 months of age with DOX treatment between 24-26 months of age, n = 9 (4 males and 5 females); age-matched non-transgenic littermates, n = 1 (12, 21 and 24 months of age each, all females), n = 4 (26 months of age with DOX treatment, 2 males and 2 females). Tg2576 mice at 20 months of age, n = 2 (1 male and 1 female).
